# Cross-beta nanostructures based on dinaphthylalanine Gd-conjugates loaded with doxorubicin

**DOI:** 10.1038/s41598-017-00332-3

**Published:** 2017-03-22

**Authors:** Carlo Diaferia, Eliana Gianolio, Teresa Sibillano, Flavia Anna Mercurio, Marilisa Leone, Cinzia Giannini, Nicole Balasco, Luigi Vitagliano, Giancarlo Morelli, Antonella Accardo

**Affiliations:** 1 Department of Pharmacy, Research Centre on Bioactive Peptides (CIRPeB), University of Naples “Federico II”, Via Mezzocannone 16, 80134 Naples, Italy; 2 Department of Molecular Biotechnologies and Health Science, University of Turin, Via Nizza 52, 10125 Turin, Italy; 3 Institute of Crystallography (IC), CNR, Via Amendola 122, 70126 Bari, Italy; 4 Institute of Biostructure and Bioimaging (IBB), CNR, via Mezzocannone 16, 80134 Naples, Italy

## Abstract

Very recently we proposed novel di- and tetra-phenylalanine peptides derivatized with gadolinium complexes as potentials supramolecular diagnostic agents for applications in MRI (Magnetic Resonance Imaging). It was observed that in very short FF dipeptide building blocks, the propensity to aggregate decreases significantly after modification with bulky moiety such as Gd-complexes, thus limiting their potential as CAs. We hypothesized that the replacement of the Phe side chain with more extended aromatic groups could improve the self-assembling. Here we describe the synthesis, structural and relaxometric behavior of a novel water soluble self-assembled peptide CA based on 2-naphthylalanine (2Nal). The peptide conjugate Gd-DOTA-L_6_-(2Nal)_2_ is able to self-assemble in long fibrillary nanostructures in water solution (up to 1.0 mg/mL). CD and FTIR spectroscopies indicate a β sheet secondary structure with an antiparallel orientation of single strands. All data are in good agreement with WAXS and SAXS characterizations that show the typical “cross-β pattern” for fibrils at the solid state. Molecular modeling indicates the three-dimensional structure of the peptide spine of aggregates is essentially constituted by extended β-sheet motifs stabilized by hydrogen bonds and hydrophobic interactions. The high relaxivity of nanoaggregates (12.3 mM^−1^ s^−1^ at 20 MHz) and their capability to encapsulate doxorubicin suggest their potential application as supramolecular theranostic agents.

## Introduction

**Figure 1:**
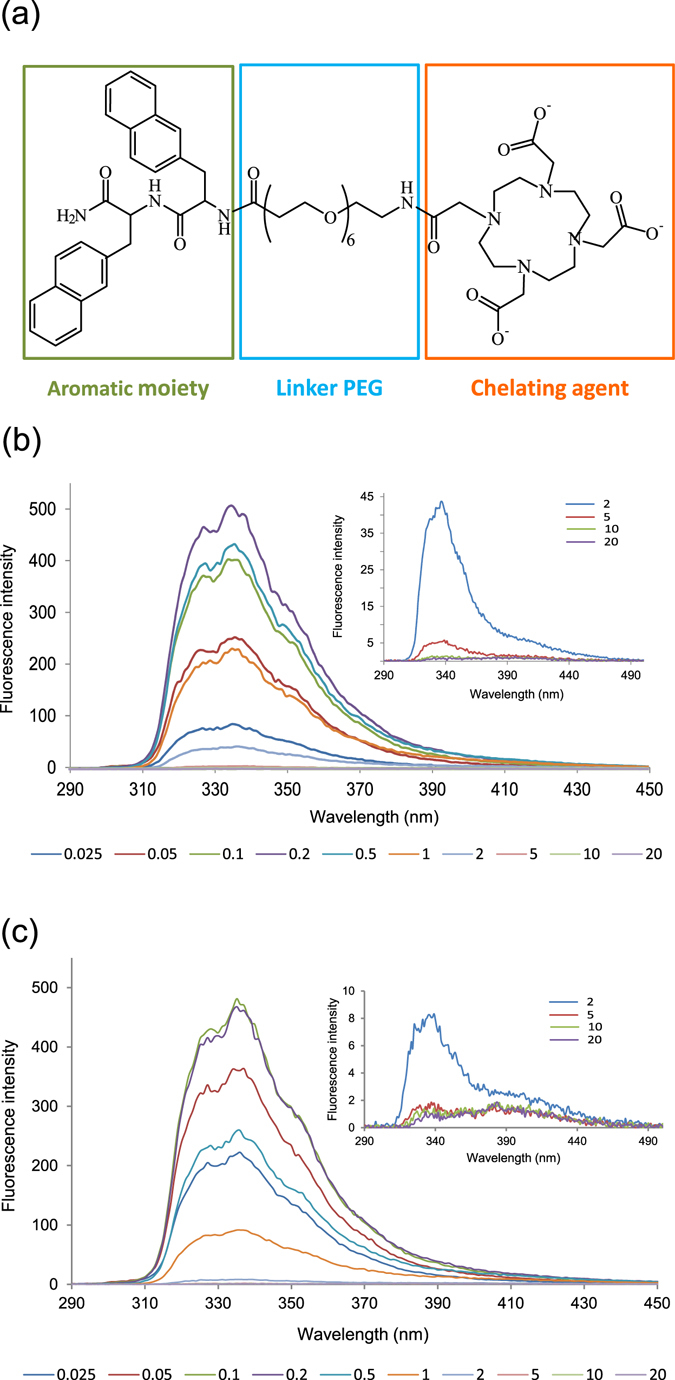
(**a**) Schematic representation of di-aromatic compounds obtained by SPPS with Fmoc chemistry. Conjugates contain an aromatic framework consisting of two 2-naphthylalanine (2-Nal) residues, an ethoxylic linker (L_6_) formed by six PEG units and bifunctional and macrociclic chelating agent (DOTA) for gadolinum (III) coordination. (**b** and **c**) Fluorescence spectra of 2Nal_2_ and Gd-2Nal_2_ in 0.025–20.0 mg/mL concentration range. Samples were excited at λ = 280 nm and recorded between 290 and 500 nm.

Since its identification as recognition motif of the Alzheimer’s β-amyloid peptide, the aromatic homodimer diphenylalanine (FF) has been largely studied for its capability to self-organize into a large variety of nanostructures (NSs) from nanotubes to nanofibrils, vesicles and organogels^1, 2^. The morphological variability of NSs structurally depends on the experimental procedure adopted for their preparation such as the polarity of the solvent^3^, the pH value^4^ or temperature^5^. In addition, chemical modifications of the aromatic homodimer, including the introduction of a thiol group or of a fluorenylmethyloxycarbonyl (Fmoc) group, can cause variations in the morphology of the aggregate^2^. The morphology of the FF-NSs seems to be determinant for their physicochemical properties. Mechanical, electrochemical and optoelectronic properties of FF-NSs^2^ leave envisage a their potential application in nanofabrication and industrial fields. However, only a few studies have been devoted, until now on their potential abilities in the biomedical field^2^. Moreover, also their applications in the diagnostic field remains unexplored, overall for the low intrinsic water solubility of these derivatives. Very recently we described the first example of supramolecular aggregates based on PEGylated cationic di-phenylalanine and tetra-phenylalanine conjugates as enhanced contrast agents (CAs) for diagnostic applications in Magnetic Resonance Imaging (MRI)^6^. These conjugates contain two (FF or F2) or four (F4) phenylalanine residues for promoting the self-assembly, a branched or linear bifunctional chelating agent such as DOTA (1,4,7,10-tetraazacyclododecane-N,N,N,N-tetraacetic acid) or DTPA (diethylenetriamine penta-acetate), for achieving the kinetically stable and thermodynamically inert coordination of the gadolinium paramagnetic ion and an ethoxylic linker at six PEG units (L_6_) between the chelating agent and the oligopeptide. In zwitterionic FF homodimer the self-assembling pathway is driven by head-to-tail hydrogen-bonding between the charged termini of neighbouring dipeptides and “T-shaped” contacts between the amino acid side-chains^7, 8^. On the contrary, in capped uncharged dipeptide FF the loss of the head-to-tail hydrogen-bonding occurs. Nevertheless, the aromatic stacking interactions allow for recovering the self-aggregation behaviour^9^. However, we observed that in very short peptide building blocks the propensity to aggregate decreases significantly after modification with bulky moiety such as Gd-complexes^6^. On the contrary F4 conjugates, with a more extended aromatic framework, keep their capability to self-aggregate giving well-structured nanofibres, also after the complexation of the gadolinium ions. These results point out that the elongation of the aromatic framework from two to four residues can represent a successful strategy to recover the aggregation propensity. As an alternative, the replacement of the Phe side chain with more extended aromatic groups could increase the self-assembling propensity of these compounds. Here, we report the synthesis of a novel peptide conjugate DOTA-L_6_-(2Nal)_2_ (Fig. 1a) obtained replacing the phenylalanine with the non-coded amino acid 2-naphthylalanine (2Nal). Both the aromatic conjugate DOTA-L_6_-(2Nal)_2_ (mentioned as 2Nal_2_) and its gadolinium complex Gd-DOTA-L_6_-(2Nal)_2_ (reported as Gd-2Nal_2_) are able to self-assemble spontaneously in water solution. The morphology of the supramolecular assemblies was evaluated with transmission electron microscopy (TEM). A deep characterization of nanostructures at the nano and atomic scale was achieved both in solution and at the solid state with SAXS/WAXS, fluorescence, ^1^HNMR, Fourier Transform Infrared (FTIR), circular dichroism (CD), and molecular modelling. The relaxivity properties of these nanostructures were studied. Moreover, fluorescence spectroscopy and ^1^HNMR studies and optical microscopy images provide the evidence for their capability to encapsulate anticancer drugs such as doxorubicin.

## Results and Discussion

### Design synthesis and fluorescence studies

The very short peptide conjugate Gd-DOTA-L_6_-F2 was previously synthesized and characterized in our previous work^6^. Physicochemical characterization pointed out that DOTA-L_6_-F2 is able to weakly self-aggregate and only before gadolinium coordination. The aggregation is mediated by π-stacking between the aromatic side chains of phenylalanine residues. Due to steric repulsion of the Gd-complex, after metal coordination, the dipeptide seems unable to keep π-π interactions. The replacement of the phenyl group with a more extended aromatic one (such as 2-naphthyl group) could in principle restore the stacking. In this perspective, we synthesized and fully characterizedDOTA-L_6_-(2Nal)_2_ (here indicated as 2Nal_2_), an analogue of the parental DOTA-L_6_-F2, in which the two phenylalanine residues were replaced with the non-coded amino acid 2-naphthylalanine (2-Nal). Peptide synthesis of 2Nal_2_ was achieved according to the standard protocols of the solid phase synthesis with Fmoc/tBu strategy, the peptide was then characterized with LC-MS (see Figure S1A) and ^1^H-NMR. After purification, the DOTA chelating agent was complexed with lanthanide metal ions, i.e. gadolinium (Gd) or lanthanum (La) for MRI or NMR studies, respectively. Nevertheless in spite of the higher hydrophobicity of the naphthyl group with respect to the phenyl one, the Gd-2Nal_2_ derivative keeps high water solubility, and the solutions remain perfectly clear up to 50 mg/mL; whereas a further increase of the concentration causes its hydrogelation. Lowering the temperature below 5 °C, fast hydrogel formation occurs yet at 20 mg/mL. The peptide conjugate Gd-2Nal_2_ shows high stability also in physiological conditions (10 mM phosphate buffer 0.9 wt.% NaCl at pH 7.4) (see Figure S2). Self-organization of the peptide conjugates at the atomic level was further characterized also in solution with fluorescence, UV-Vis, ^1^HNMR, CD and FTIR spectroscopies. In Fig. 1b and c are reported fluorescence spectra of 2Nal_2_ and Gd-2Nal_2_ at several concentrations (from 0.025 to 20.0 mg/mL) obtained by exciting the samples at 280 nm, which corresponds to the wavelength of absorption for the 2-naphthylalanine (Figure S1B). From ispection of Fig. 1b, the typical emission spectrum of the 2-naphthyl group with three maxima at 325, 340 and 355 nm, respectively, can be detected. The fluorescence intensity of 2Nal_2_ (see Fig. 1b) increases gradually in the range of concentration between 0.025 and 0.2 mg/mL. After this concentration a progressive intensity decrease, attributable to the stacking of the aromatic rings, occurs. Since 2.0 mg/mL, the fluorescence intensity is very low. From ispection of the inset in Fig. 1b a weak peak, that is centered between 380–430 nm and indicative of excimer formation, is visible. These results suggest that the peptide derivative begins to undergo self-aggregation phenomena at 0.2–0.5 mg/mL (150–375 μM). However, the appearance of the excimer peak above 2.0 mg/mL leaves us to hypotize that very stable aggregates can be formed above 2.0 mg/mL. A similar behaviour was observed afterthe gadolinium coordination (see Fig. 1c). However in Gd-2Nal_2_, both the decrease of the fluorescence intensity (0.1 mg/mL) and the appearance of the excimeric peak (1.0 mg/mL) were observed at lower concentration with respect to 2Nal_2_, thus confirming that the neutralization of the negative charges on the chelating agent, after the Gd-coordination, induces a better self-assembling in these peptide conjugates. Determination of the critical aggregation concentration (CAC) value of Gd-2Nal_2_ was carried out using a fluorescence-based method, in which ANS was used as fluorescent probe. This dyeis completely silent at the fluorescence in aqueous solution, but emits in the range 460–480 nm when is surrounded by a hydrophobic environment^10^. A solution of ANS in cuvette (20 μM) was titrated with Gd-2Nal_2_ and the fluorescence intensities maximum measured at 470 nm have been plotted in Figure S3. CAC value, determined from the graphical break-point, is ∼6.70 10^−4^ M (0.86 mg/mL). This value is 10-fold higher than the CAC value (5.1·10^−5^ M, 0.076 mg/mL) previously found for tetraphenylalanine Gd-complex Gd-DOTA-L_6_-F4. This consideration suggests that the replacement of the phenyl ring with the naphthyl one allows to increase the aggregation propensity with respect to diphenylalanine conjugates (Gd-DOTA-L_6_-F2). However from the comparison of CAC values (Gd-DOTA-L_6_-F2 > Gd-DOTA-L_6_-Nal_2_ > Gd-DOTA-L_6_-F4) it emerges that the increase of the number of aromatic residues^6^ causes a better aggregation with respect to the replacement of Phe with more extended aromatic group.

### NMR studies

This trend was also confirmed by ^1^HNMR measurements (see Figure S4). We have recently analyzed the conformational properties of DOTA-L_6_-F4 in aqueous solution by mono- and multi-dimensional, homo- and hetero-nuclear NMR techniques^11^. In the present work we have used a similar approach and thus implemented 1D [^1^H] and 2D [^1^H, ^1^H] NMR spectroscopy to study two different compounds: DOTA-L_6_-F2 and 2Nal_2_ analogue in absence and in presence of the DOTA coordinated metal ion lanthanum (III), La. We used lanthanum (III) since it gives rise to a NMR “easy to handle” diamagnetic complex. Both DOTA-L_6_-F2 and 2Nal_2_ present low aggregation propensities. The process of resonance assignments of the two molecules could be easily achieved at a concentration equal to 1 mM (see Supplemental Material Tables S1, S2 and Figure S5). For the DOTA-L_6_-F2 relevant changes in the spectra, indicating occurrence of potential aggregation processes, take place at a concentration higher than 1.2 mg/mL (Figure S4a-left panel). In detail by comparing spectra recorded at 0.3 mg/mL and 5.0 mg/mL chemical shift changes appear relevant in the spectral regions between 8.0 and 8.2 ppm, where signals from HN amide protons are present and between 3.0 and 3.5 ppm where several peaks overlap (Table S1). However, even at the highest investigated concentrations, peaks in the NMR spectra remain rather sharp thus pointing towards formation of small size aggregates. Once complexed with La (III) the DOTA-L_6_-F2 presents similar weak aggregation propensity (Figure S4b-left panel). For 2Nal_2_ in the absence of coordinated metal ions, from 0.1 mg/mL till 5.0 mg/mL, we cannot reveal significant aggregation as the signals in the NMR spectra do not become broad or change their chemical shifts (Figure S4a-rigth panel). Once La (III) is inserted in 2Nal_2_ the aggregation tendency increases and line broadening starts to affect the spectra thus indicating formation of aggregated species already at 0.8 mg/mL concentration; at 2.5 mg/mL concentration extensive line broadening causes partial signal loss thus indicating the presence of larger aggregates (Figure S4b-right panel). Aggregation as witnessed by line broadening is affecting all signals in the NMR spectrum of 2Nal_2_ (See for instance the H_N_ amide and aromatic protons regionin Figure S4b-right panel). This different aggregation property in presence and absence of the metal ion likely indicates that La (III) by neutralizing DOTA negative charges is favoring intermolecular interaction by lowering electrostatic repulsions. Moreover, NMR data show that La-2Nal_2_ has a higher tendency to aggregate with respect to La-DOTA-L_6_-F2; this outcome can be easily explained by the larger aromatic patches present in the former molecule which are likely favoring larger intermolecular π-stacking interactions. These data indicate consequently that the Gd-2Nal_2_ may work better as a potential MRI contrast agent than Gd-DOTA-L_6_-F2.

### Secondary structure characterization

**Figure 2:**
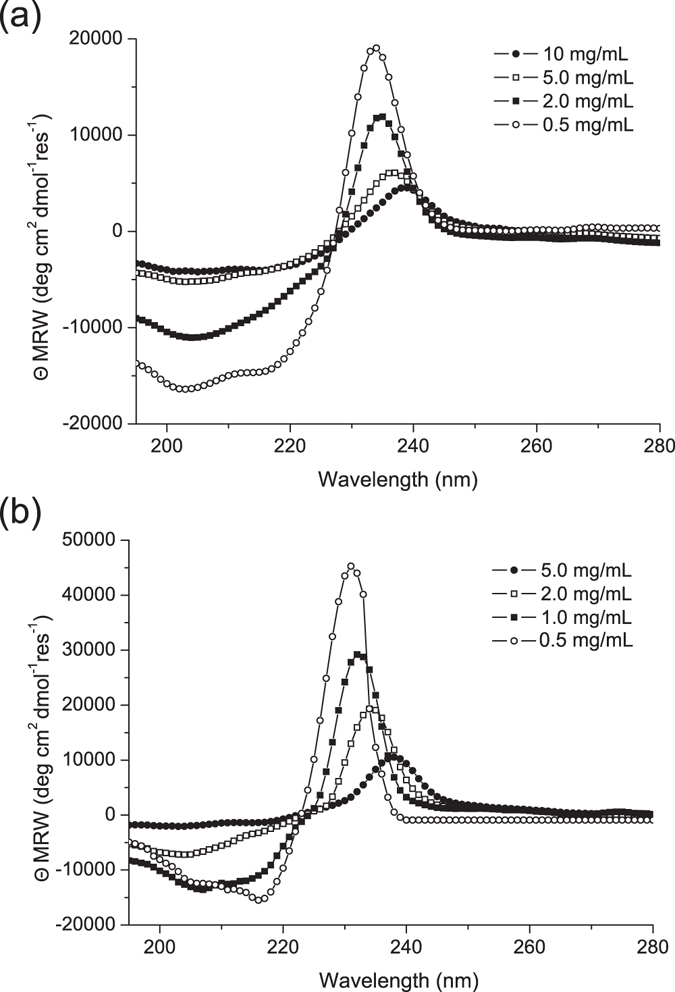
Selected Far-UV CD spectra of 2Nal_2_ (**a**) and Gd-2Nal_2_ (**b**) in a concentration range of 0.5–20.0 mg mL^−1^. Spectra are recorded between 280 and 195 nm.

**Figure 3:**
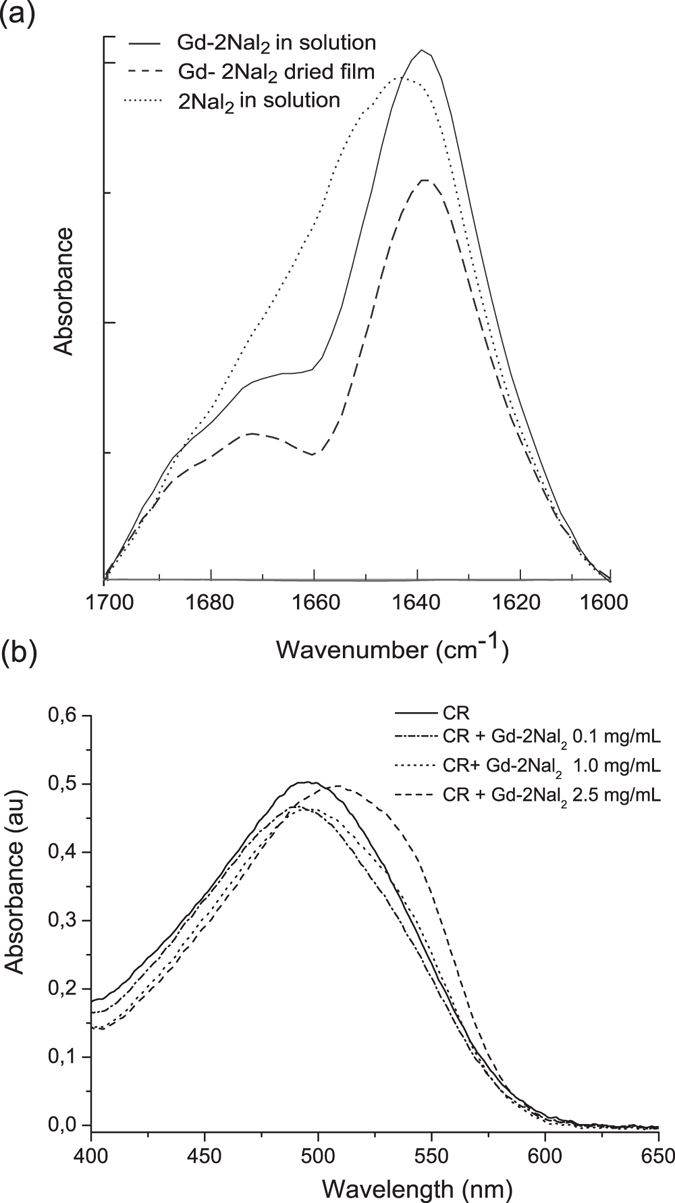
(**a**) FTIR spectra of 2Nal_2_ and Gd-2Nal_2_ in the amide I region at 5.0 mg/mL. (**b**) UV-Vis spectra of 2Nal_2_ and Gd-2Nal_2_, stained with Congo Red at 0.1 and 1.0 mg/mL. The spectrum of Congo Red is also reported for comparison.

Secondary structure assumed by 2Nal_2_ and Gd-2Nal_2_, was investigated by Circular Dichroism (CD) and by Fourier Transform Infrared (FTIR) spectroscopies. CD spectra, reported in Fig. 2a and b, were recorded both below and above the self-aggregation concentration. Independently from the coordination of the metal, 2Nal_2_ peptide shows a similar dichroic signature: at 0.5 mg/mL, there are two minima around 205 and 218 nm and a maximum at 232 nm. This dichroic signature is perfectly identical and symmetric to that previously observed for tetra-phenylalanine conjugates^6^. The two minima are attributable to the stacking between the 2-naphthyl aromatic rings, whereas the maximum is typically observed in presence of β-structure. Above this concentration value a progressive red-shift of the maximum (up to 340 nm), as function of the peptide concentration, occurs. This spectral shift is accompanied by the decrease of the maximum and by the presence of an isosbestic point. Both the shift and the decrease of the maximum indicate the formation in solution of nanostructures with a dominant β-sheet arrangement. Further information on the secondary structural organization of the peptide conjugates was achieved using FTIR spectroscopy in the region of the amide I vibrational band (1600–1700 cm^−1^). FTIR spectra of 2Nal_2_ and Gd-2Nal_2_ at 5.0 mg/mL in the amide I region are reported in Fig. 3a. Spectrum of Gd-2Nal_2_ as dried film is also reported. Gd-2Nal_2_ FTIR spectra, both at the solid state and in solution, show a dominant peak around 1638 cm^−1^, expected for β-sheet formation. In addition, both spectra have a secondary peak around 1680 cm^−1^, that is normally observed for antiparallel orientation of the β-sheet. By comparing these spectra with spectrum of 2Nal_2_, it appears a very broad peak at 1642 cm^−1^.

### Congo Red spectroscopic assay

The high tendency of Gd-2Nal_2_ to self-aggregate in amyloid-like fibrillary nanostructures in solution was confirmed by Congo Red (CR) staining assay. CR is a well-known azoic dye, used as indicator of the occurrence of amyloid like fibrils^12^. CR alone exhibits in UV-Vis a typical absorbance maximum at 490 nm (see Fig. 3b). When incubated with fibrillary aggregates, its spectral profile changes and a shift of the CR band from 490 to 540 nm is expected. In our experiment, depicted in Fig. 3b, CR UV-Vis maximum undergoes a clear shift after incubation with Gd-2Nal_2_ at 2.5 mg/mL. Instead, any variation or a slight shift can be evidenced for CR incubated with 0.1 and 1.0 mg/mL, respectively.

### Materials characterization and molecular modelling

**Figure 4:**
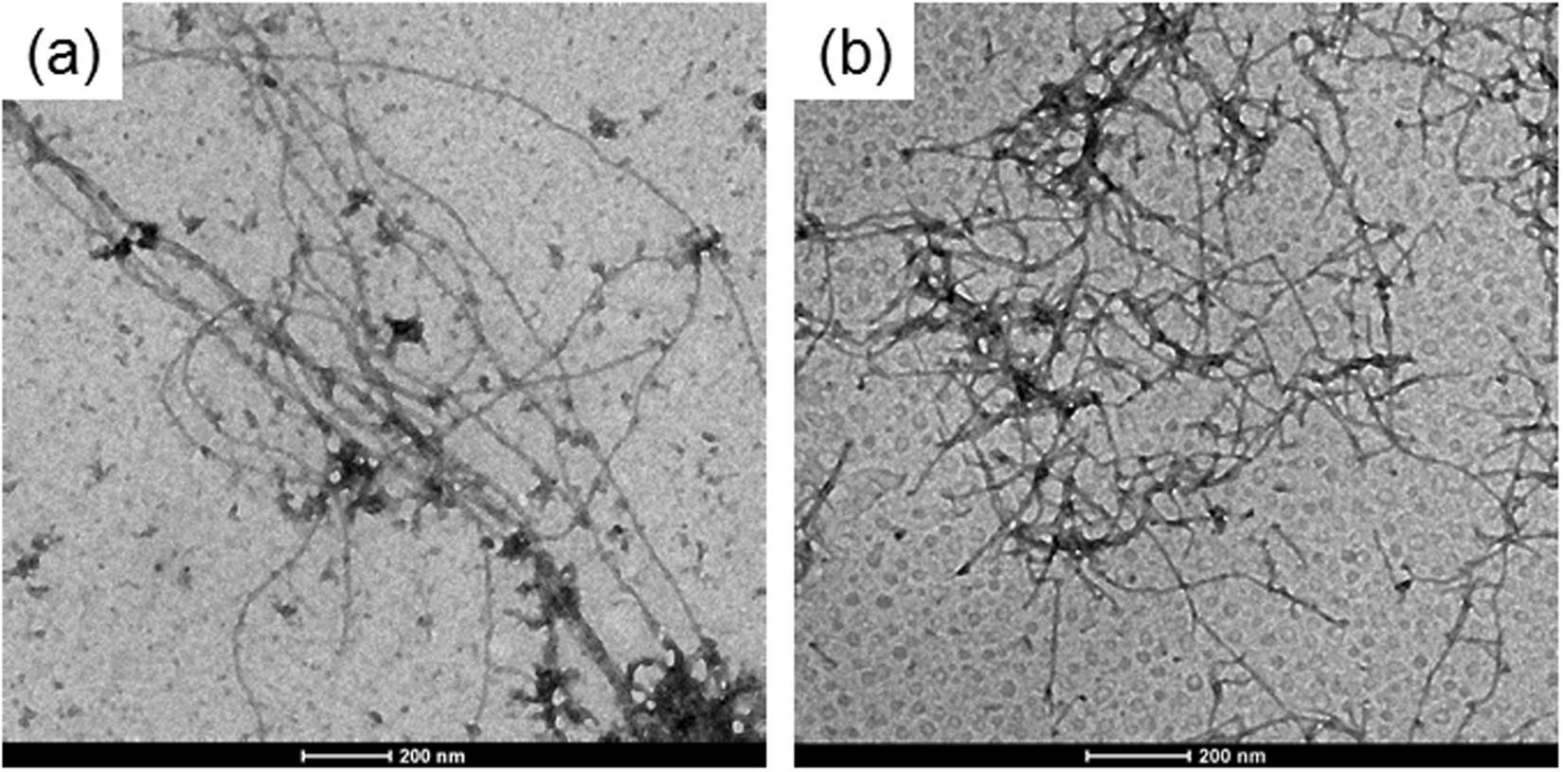
Selected TEM images for 2Nal_2_ (**a**) and Gd-2Nal_2_ (**b**) at 5.0 mg/mL.

**Figure 5:**
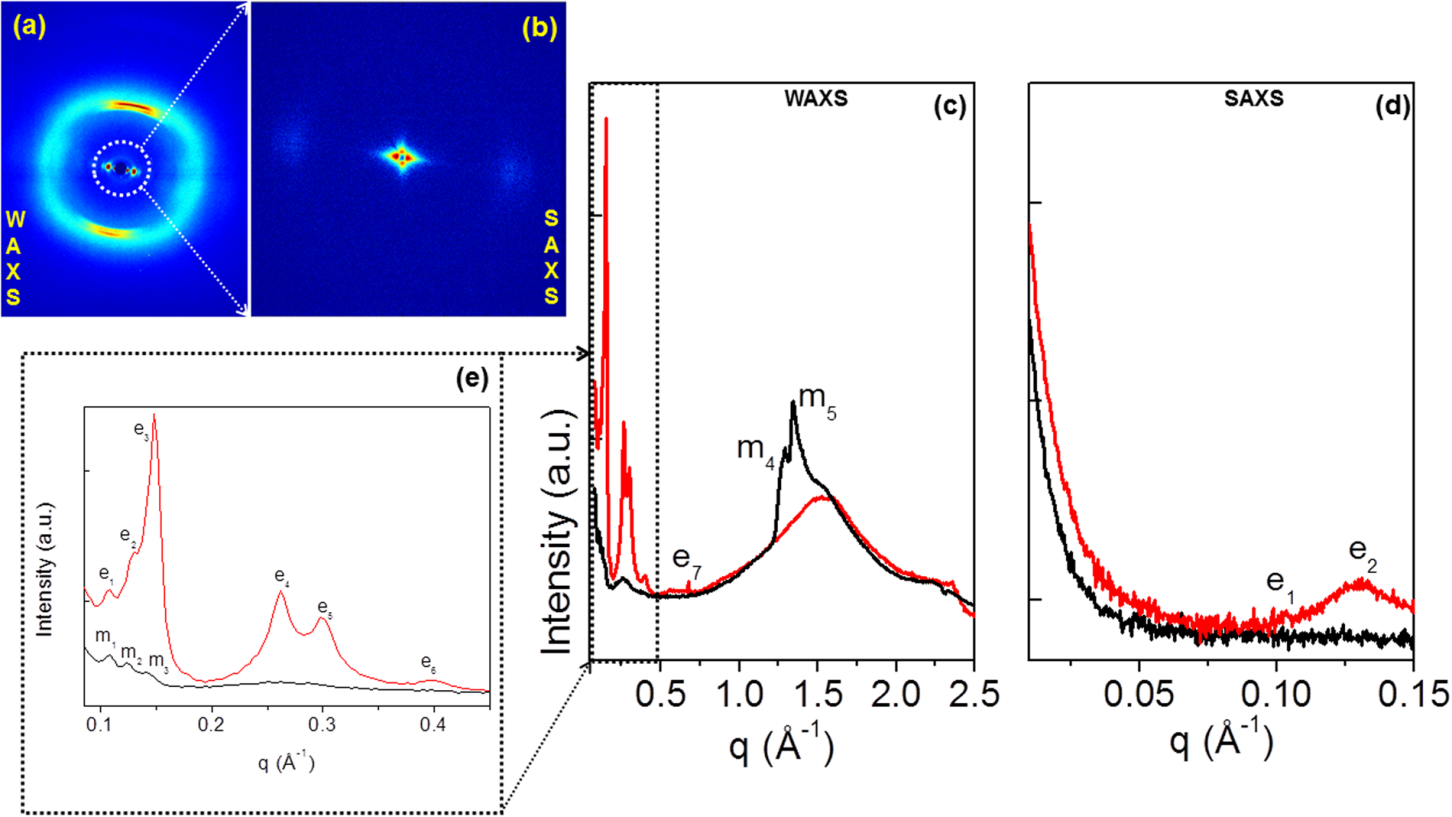
2Nal_2_ fiber at solid state: (**a**,**b**) 2D WAXS and SAXS data; (**c**,**d**) 1D WAXS and SAXS profiles (red and black corresponding to the meridional and equatorial directions) as obtained once the corresponding 2D WAXS (**a**) and SAXS (**b**) data are centered, calibrated and radially folded; the inset in panel (**d**) refers to the equatorial e2 reflection, with an estimated FWHM (e2-2Nal_2_) = 19°; (**e**) expanded WAXS region marked by a dotted rectangle in (**c**).

**Figure 6:**
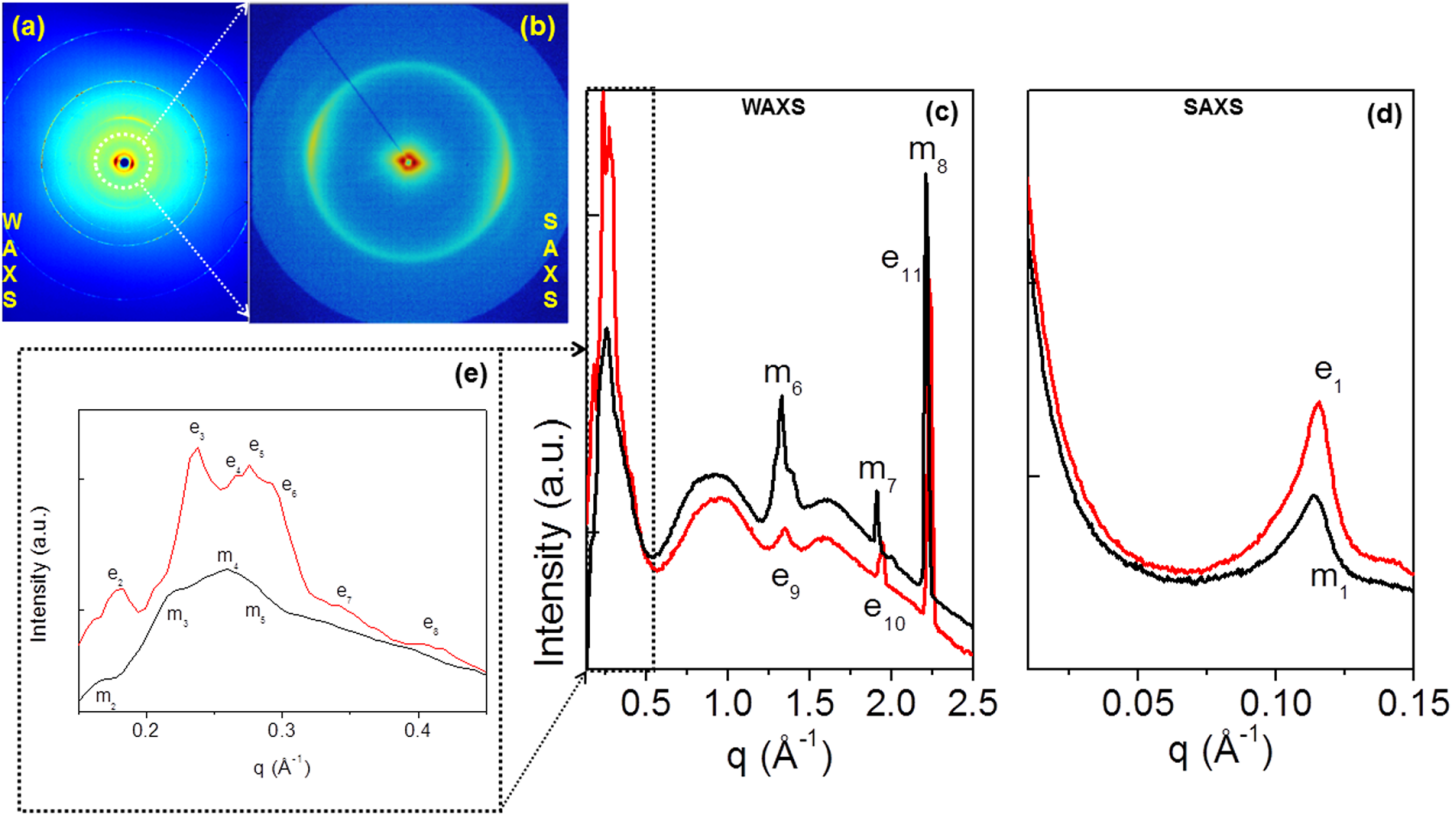
Gd-2Nal_2_ fiber at solid state: (**a**,**b**) 2D WAXS and SAXS data; (**c**,**d**) 1D WAXS and SAXS profiles (red and black corresponding to the meridional and equatorial directions) as obtained once the corresponding 2D WAXS (**a**) and SAXS (**b**) data are centered, calibrated and radially folded; the inset in panel (d) refers to the equatorial e1 reflection, with an estimated FWHM (e1-Gd-2Nal_2_) = 27°; (**e**) expanded WAXS region marked by a dotted rectangle in (**c**).

**Figure 7:**
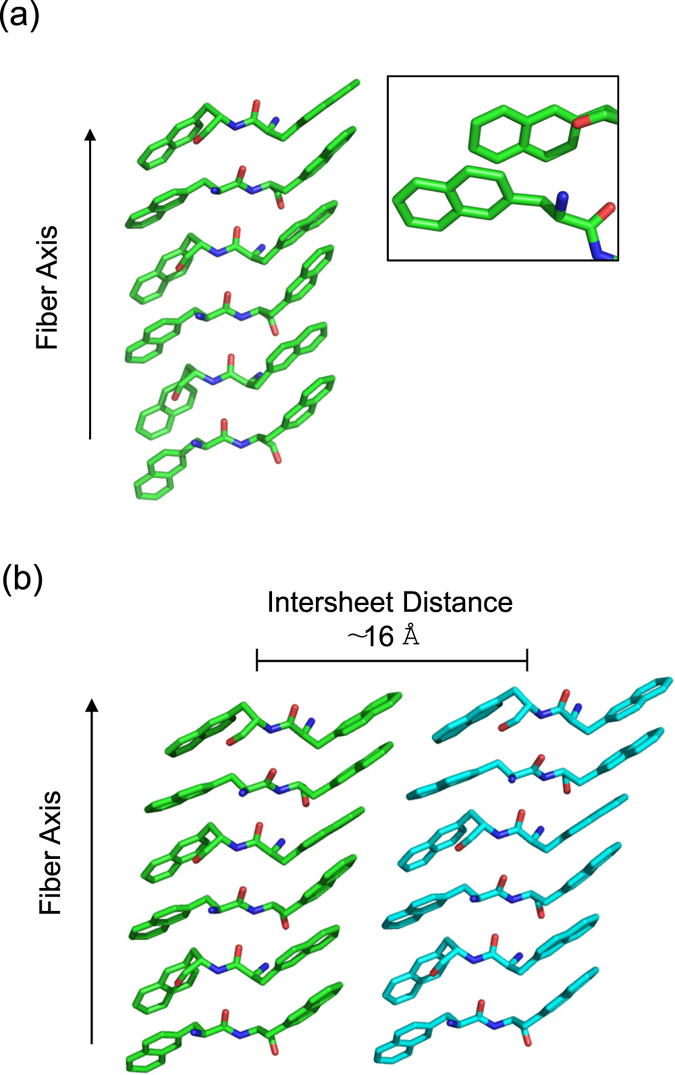
Three-dimensional model of the assembly formed by the peptide moiety of 2Nal_2_/Gd-2Nal_2_. In panel (**a**) the cross β-structure of a single β-sheet is reported. In the inset the extensive hydrophobic interactions established by Nal side chains are highlighted. The possible direct interactions between two facing β-sheets is reported in panel (**b**).

Self-assembled peptide conjugate at 5.0 mg/mL, as free base or as metal complex, was initially characterized using TEM. TEM images reported in Fig. 4a and b demonstrate the assembling of both 2Nal_2_ and Gd-2Nal_2_ in long fibrillary nanostructures. On the other hand, TEM images of Gd-DOTA-L_6_-F2 do not show any supramolecular aggregate (data not shown). To further analyze the supramolecular morphology of 2Nal_2_ and Gd-2Nal_2_ at the micro and nano scale, WAXS and SAXS measurements were performed on dried fibres prepared according to the stretch frame method^13^. Figures 5a,b and 6a,b present the two-dimensional (2D) WAXS and SAXS patterns collected on the 2Nal_2_ and Gd-2Nal_2_ samples, respectively. The 2D patterns, once centered, calibrated and radially folded into 1D profiles, are shown in Figs 5c,d and 6c,d, respectively. The low-q region of each WAXS pattern is displayed separately in Figs 5e and 6e to better visualize the diffraction peaks. The red and black WAXS/SAXS profiles correspond to the meridional and equatorial directions marked by the red arrows in panels (b). The position of most intense meridional and equatorial diffraction peaks are reported in Table 1. Both fibers present the classic cross-β fiber 2D WAXS diffraction pattern with the β-strands distance at d_β1_ = 4.7 ± 0.3 Å along the meridional direction (fiber axis). The 2D SAXS patterns display a clear diffraction structure. The most intense peaks lay along the equatorial direction, for both samples. The presence of a diffraction pattern both in SAXS and in WAXS is fingerprint of a hierarchical organization of the molecules in fibers from the atomic to the nanoscale. The most intense SAXS peak in the Gd-2Nal_2_ sample, labelled as e1 in Fig. 6d, corresponding to d = 57 Å, almost disappears in the 2Nal_2_, where the most intense SAXS peak occurs at d = 48 Å (e2 equatorial peak in Fig. 5d). We estimated the full-width-at-half-maximum along the azimuth angle for the e1 and e2 peaks in the insets of Figs 5d and 6d, which are FWHM (e1-Gd-2Nal_2_) = 27° and FWHM (e2-2Nal_2_) = 19°, respectively. Furthermore, for both aggregates intense equatorial peaks were observed in WAXS experiments (d range 21–25 Å). Although peaks in this region occur in both 2Nal_2_ and Gd-2Nal_2_ samples their location and intensity are not identical. This observation suggests that the metal complexation of 2Nal_2_ induces some structural rearrangements. Altogether, SAXS and WAXS experiments provide some interesting clues on the organization of the peptide spine of these assemblies at different structural level. In particular, (a) the meridional peak at 4.7 Å atomic distance clearly indicates a cross-β structure in which the β-strands run perpendicular to the fiber axis, (b) the equatorial reflections at 21–25 Å likely represent regularities within the inter-sheet distances, and (c) the peaks at d = 48 and 57 Å provide some preliminary information on the nanofiber organization. In this framework, we performed molecular modeling analyses to gain insights into the atomic structure of the peptide moiety of these assemblies using the programs Coot and Pymol^14, 15^. Accordingly, we generated antiparallel cross-β models, which were stabilized by four inter-strand hydrogen bonds, to check whether Nal side chains could contribute to the stabilization of this motif. In particular, we evaluated the interactions between Nal side chains of consecutive β-strands within the cross β-sheet by considering all its possible rotameric states. We found combinations of rotameric states that maximized hydrophobic interactions between side chains of consecutive strands (Fig. 7a). Then, we evaluated how different β-sheets could laterally interact. We found that optimal interactions between the Nal side chains of facing β-sheets could be established when the inter-sheet distance was about 16–17 Å (Fig. 7b). Notably, although as minor peaks, equatorial reflections with this spacing were observed for both samples (Figs 5e and 6e). However, as mentioned above, the largest equatorial peaks that likely correspond to inter-sheet distances present spacings with d = 21–25 Å. This observation suggests that there are multiple ways in which β-sheets interact in these assemblies and that other species may mediate these inter-sheet interactions. In this scenario, it cannot be excluded that the hydrophobic rings of individual Nal molecules intercalate within the hydrophobic surfaces of facing β-sheets (Figure S6). In conclusion, our observations suggest that the basic structural elements of these assemblies is a cross β-sheet that is stabilized by both the hydrogen bonding and hydrophobic interactions established by Nal aromatic side chains. The larger size of the Nal hydrophobic side chain compared to Phe makes dipeptide cross β-sheets formed by the former residues stable enough to generate well-defined aggregates at microscale level. Finally, individual β-sheets can then mutually interact in different ways to generate the structural variability of the lateral packing observed in these aggregates.

**Table 1:** q positions, and corresponding d = 2π/q values, of meridional and equatorial reflections for the 2-Nal and Gd-2Nal_2_ fibers.

Label	2-Nal		Gd-2Nal_2_	
	q (Å^−1^)	d (Å)	q (Å^−1^)	d (Å)
e1	0.11	57	0.11	57
e2	0.13	48	0.18	35
e3	0.15	42	0.24	26
e4	0.26	24	0.26	24
e5	0.3	21	0.28	22.5
e6	0.4	16	0.29	22
e7	—		0.34	18.5
e8	—		0.40	16
e9	—		1.35	4.5
e10			1.94	3
			2.23	2.8
m1	0.11	57	0.11	57
m2	0.125	50	0.17	37
m3	0.14	45	0.22	28.5
m4	1.28	5	0.26	24
m5	1.34	4.7	0.28	22.5
m6	—		1.32	4.75
m7	—		1.9	3.3

The error on the d values amounts to ±0.3 Å.

### Water proton relaxation measurements

**Figure 8:**
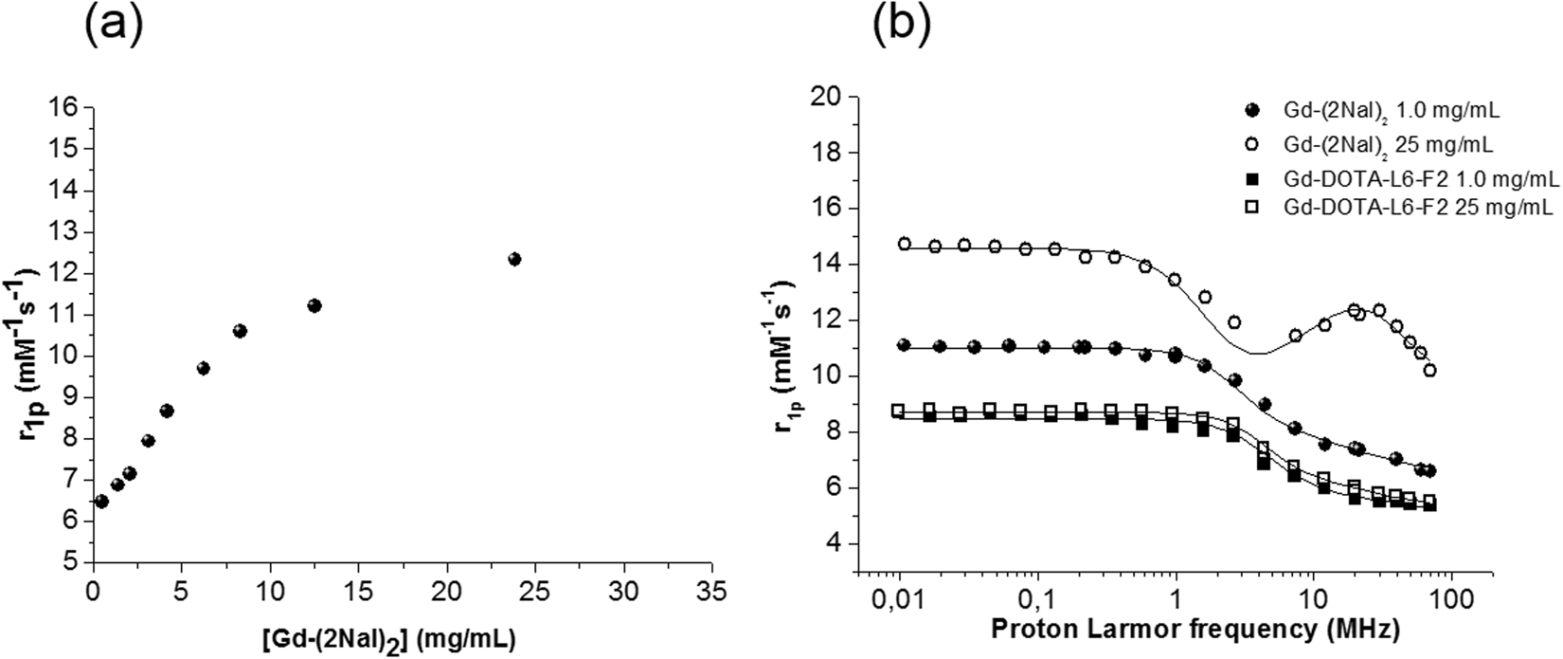
(**a**) Longitudinal proton relaxivity of Gd-DOTA-L_6_-(2Nal)_2_ measured at 21.5 MHz (0.5 T) and 298 K as a function of the concentration of the Gd-complex. (**b**) NMRD profiles of Gd-DOTA-L_6_-(2Nal)_2_ (1.0 mg/mL (●) and 25.0 mg/mL (○)) compared to those of Gd-DOTA-L_6_-F2 (1.0 mg/mL (■) and 25.0 mg/mL (□)). Experimental data points were measured on aqueous solutions of the Gd-complexes at 298 K. The data refer to 1 mM concentration of the paramagnetic complexes.

The paramagnetic relaxivity (r_1p_) of Gd (III) complexes is given by:

1 $${r}_{{1}p}\propto \frac{{1}}{{T}_{{1}M}^{H}}=K\,f({\tau }_{R})$$

where *K* is a constant related to the strength of the dipolar interaction between the protons and the electronic magnetic moment, and τ_*R*_ is the correlation time associated with the reorientation of the Gd-H magnetic vector. This means that slowly moving high molecular weight systems are endowed with higher relaxivity than rapidly reorienting low molecular weight ones. In the case of self assembling Gd-containing systems, the extent of aggregation may be evaluated through the measure of the water proton longitudinal relaxation rate of its aqueous solution. In Fig. 8 are reported the NMRD (Nuclear Magnetic Resonance Dispersion) profiles of Gd-2Nal_2_ acquired at 21.5 MHz (0.5 T) and 298 K as a function of the complex concentration. The enhancement in relaxivity indicates that Gd-2Nal_2_ starts to self-aggregate at very low concentration (ca. 1.0 mg/mL), confirming the values determined by fluorescence spectroscopy and ^1^H-NMR. At concentrations higher than 10–15 mg/mL the relaxivity assumes an almost stable value which corresponds to the system in the completely aggregated form. By analyzing the profile of the relaxivity data as a function of the applied field strength (NMRD - Nuclear Magnetic Relaxation Dispersion) it is possible to obtain an accurate determination of the reorientational correlation time (τ_*R*_)^16^, that is strictly related to the molecular size of the investigated system. NMRD profiles were acquired below (black circles) and above (white ones) the CAC values determined by ANS titration and ^1^HNMR. In Fig. 8b the NMRD profiles of Gd-2Nal_2_ at 1.0 and 25.0 mg/mL concentrations are reported and compared to those of the corresponding di-phenylalanine conjugate Gd-DOTA-L_6_-F2 at the same concentrations. Data were analyzed using the Solomon-Bloembergen-Morgan model^17, 18^, considering one water molecule in the inner coordination sphere (q = 1) and fixing the exchange lifetime (τ_M_) to a reliable value (700 ps) on the basis of those previously reported for mono-amido DOTA derivatives^19–22^. The quantitative analysis of the NMRD profile of the aggregated form was not satisfactory when the simple inner- outer-sphere model was used, so the Lipari-Szabo approach was used for the description of the rotational dynamics. This model allows one to take into account the presence ofa certain degree of internal rotation superimposed on the overall tumbling motion^23, 24^. These two types ofmotion, a relatively fast local rotation of the coordination cage about the linker to the peptide scaffold superimposed on the global reorientation of the system, are characterized by different correlation times: τ_R_^l^ and τ_R_^g^, respectively. The degree of correlation between global and local rotations is given by the parameter S^2^, which takes values between 0 (completely independent motions) and 1 (entirely correlated motions). Both the shape of the profiles and the τ_R_ values determined from the fitting of the experimental results (Table 2) validate the hypothesis of the formation of aggregates for Gd-2Nal_2_ at 25.0 mg/mL. On the contrary, the system is in the monomeric state when it is dissolved in solution at 1 mg/mL concentration. In the case of Gd-DOTA-L_6_-F2, both qualitative and quantitative analysis confirm the inability of the system to self-aggregate even at high concentration. The quantitative analysis of the NMRD profiles indicates that both the local and the global rotational correlation times (Table 2) are significantly increased as compared to the τ_R_ value of the Gd-complex in the monomeric form, whereas the S^2^ value is relatively low (S^2^ = 0.23), suggesting great flexibility of Gd-coordination cage around the oxoethylenic linker.

**Table 2:** Relaxometric parameters extracted from the fitting of NMRD profiles reported in Fig. 8 ^[a]^.

System		r_1p_ (mM^−1^ s^−1^)	Δ^2^ (s^−2^)^[b]^	τ_V_ (ps)^[c]^	τ_R_ (ps)^[e]^		
Gd-DOTA-L_6_-(2Nal)_2_	1 mg/mL	7.3	1.02 × 10^19^	50	184		
	25 mg/mL	12.3	8.10 × 10^18^	43	τ_R_^l^	τ_R_^g^	S^2^
					423	4800	0.23
Gd-DOTA-L_6_-F2	1 mg/mL	5.7	2.97 × 10^19^	28	120		
	25 mg/mL	6.0	2.28 × 10^19^	35	127		

^[a]^On carrying out the fitting procedure, some parameters were fixed to reasonable values: r_Gd-H_ (distance between Gd and protons of the inner sphere water molecule) = 3.1 Å; a (distance of minimum approach of solvent water molecules to Gd^3+^ ion) = 3.8 Å; D (solvent diffusion coefficient) = 2.2∙10^−5^ cm^2^ s^−1^. ^[b]^Squared mean transient zero-field splitting (ZFS) energy. ^[c]^Correlation time for the collision-related modulation of the ZFS Hamiltonian. ^[d]^Electronic relaxation time at zero field (calculated as 1/τ_s0_ = 12Δ^2^ × τ_v_). ^[e]^Reorientational correlation time.

### Doxorubicin loading and release

In order to prepare a potential theranostic agent, we assayed to incorporate cytotoxic doxorubicin (DOX) model drug in the Gd-2Nal_2_ paramagnetic nanostructures. DOX is an established anticancer agent clinically effective for the treatment of many cancertypes (breast and ovarian cancers). The incorporation and release of DOX into nanostructures was estimated by fluorescence spectroscopy (Figures S7 and S8). The addition of increasing amounts of the peptide conjugate to the DOX solution causes a progressive decrease of the fluorescence intensity of DOX (Figure S7a). This quenching effect is attributable to the electrostatic interaction between the aromatic ring of the anthracycline with the naphthylalanine one. By plotting the fluorescence intensity in the maximum at 590 nm as function of the Gd-2Nal_2_ concentration (Figure S7b) we estimated that g_Drug_/g_Peptide_ is 0.028 (∼60 μg of DOX for ∼2.0 mg of peptide). The leakage assay, reported in Fig. 8 indicate a very low efflux of DOX across the peptide aggregate (∼6% of drug released after 72 h).

**Figure 9:**
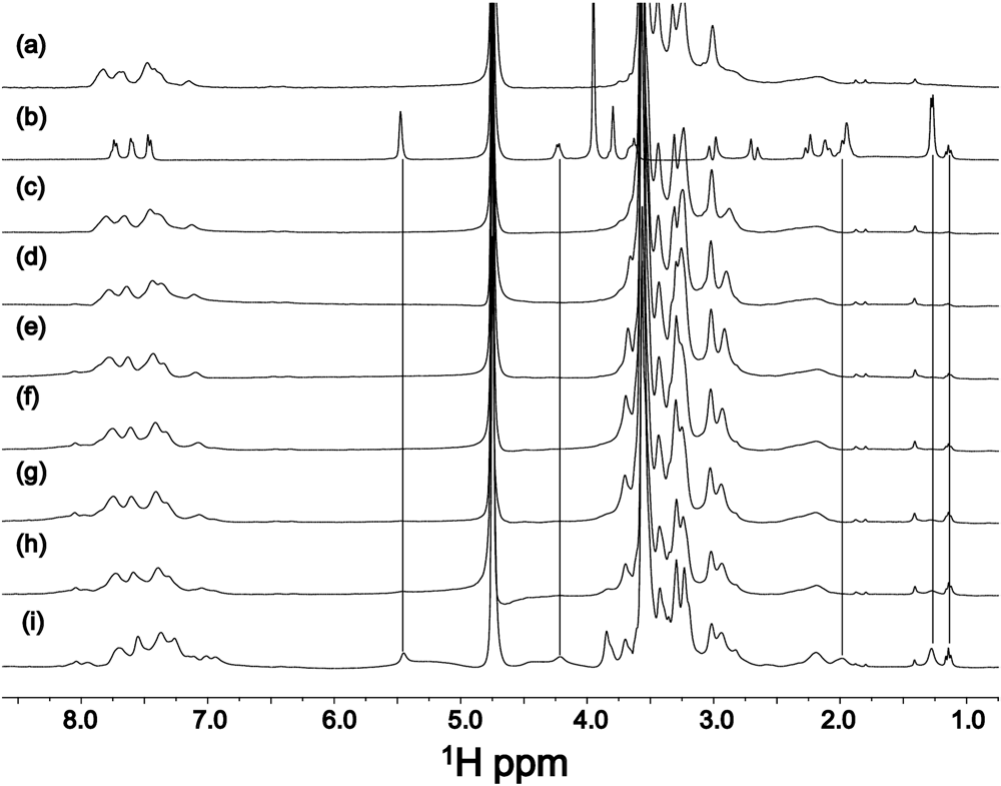
1D ^1^H NMR spectra acquired at 298 K of (**a**) La-(2-Nal)_2_ at 2.5 mg/mL, (**b**) DOX 0.5 mg/mL, (**c**) La-(2-Nal)_2_ at 2.5 mg/mL + DOX 0.05 mg/mL, (**d**) La-(2-Nal)_2_ at 2.5 mg/mL + DOX 0.1 mg/mL, (**e**) La-(2-Nal)_2_ at 2.5 mg/mL + DOX 0.2 mg/mL, (**f**) La-(2-Nal)_2_ at 2.5 mg/mL + DOX 0.3 mg/mL, (**g**) La-(2-Nal)_2_ at 2.5 mg/mL + DOX 0.4 mg/mL, (**h**) La-(2-Nal)_2_ at 2.5 mg/mL + DOX 0.5 mg/mL, (**i**) La-(2-Nal)_2_ at 2.5 mg/mL + DOX 1 mg/mL. In the lowest spectrum a few peaks arising from doxorubicin appear, as indicated by the black lines.

**Figure 10:**
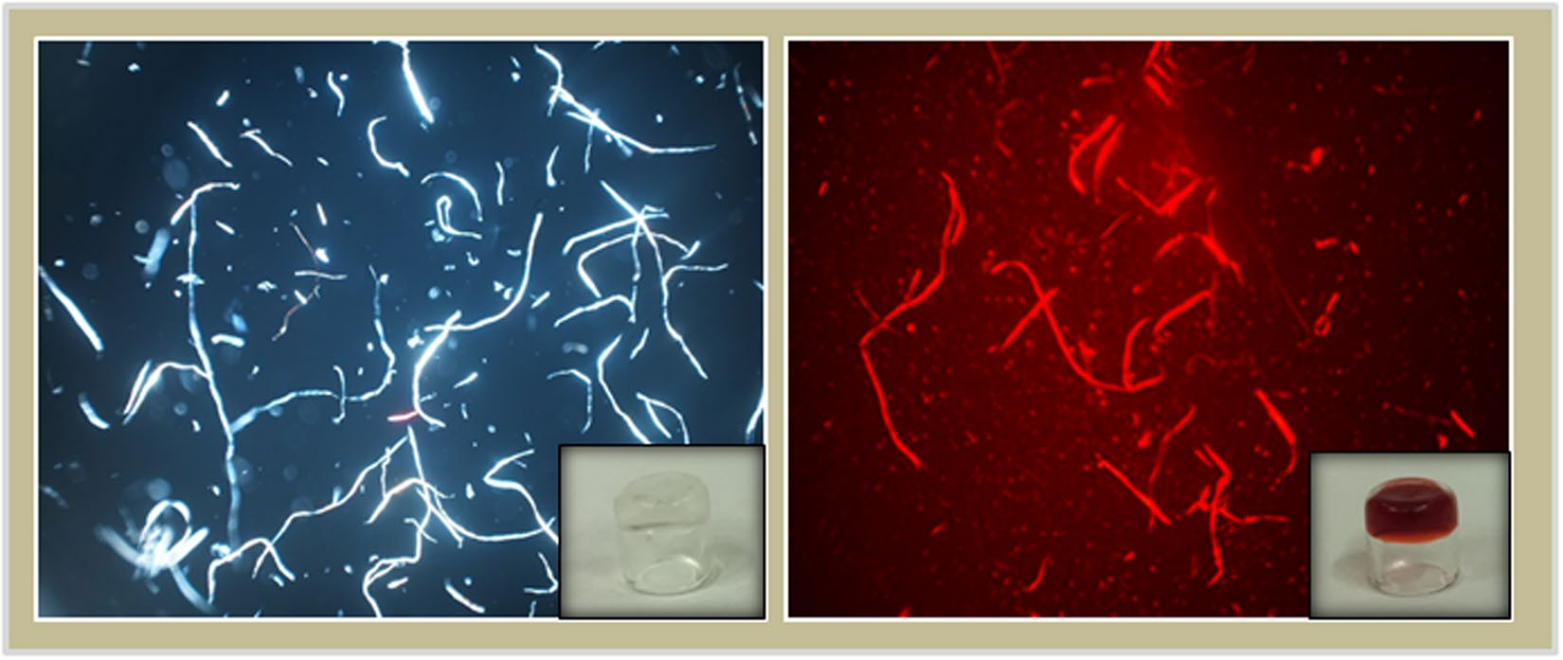
Polarized optical microscopy image of 1-cm vials containing (**a**) Gd-2Nal_2_ and (**b**) DOX encapsulating Gd-2Nal_2_ hydrogels. Images areobserved with a Nikon microscope.

The ability of 2Nal_2_ aggregates to encapsulate DOX was also checked by running 1D [^1^H] spectra of La-2Nal_2_ (at 2.5 mg/mL)at increasing DOX concentrations (Fig. 9). At a concentration equal to 2.5 mg/mL, as indicated above, La-2Nal_2_ has formed relatively large aggregates (Fig. 9a). In absence of La-2Nal_2_, doxorubicin NMR spectrum in water (0.5 mg/mL concentration) contains sharp peaks (Fig. 9b). In presence of La-2Nal_2_ aggregates, doxorubicin signals are large and can be barely recognized in the spectrum, thus indicating the encapsulation of the drug inside the aggregates (Fig. 9c–i). The extensive line broadening and loss of signal is caused by the improved relaxation rate reflecting an increase of molecular weight with respect to the free doxorubicin. Once all the pores inside the supramolecular aggregates of La-2Nal_2_ are saturated, the drug starts to be released and this occurs at a concentration of doxorubicin close to 1.0 mg/mL (Fig. 9i). At this drug concentration signals are still affected by line broadening thus indicating that most of the molecules are still entrapped inside the aggregates. Incorporation of DOX in Gd-2Nal_2_ nanostructures was further examinedby optical microscopy. Figure 10 shows polarizing optical micrographs (POM) of empty Gd-2Nal_2_ nanostructures compared to DOX filled Gd-2Nal_2_ ones. Polarized light interacts strongly with both the birefringent samples, generating contrast with the background.

## Conclusions

Diphenylalanine peptide remains the most largely studied peptide building block until now for self-aggregating peptide materials. Their capability to self-aggregate can considerable decrease hereafter the modification with bulky moiety such as Gd-complexes. This consideration is particularly true for the cationic FF peptide, in which the replacement of the carboxylic group at the C-terminus with the amidic one causes the loss of the head-to-tail hydrogen-bonding. In CAs based FF (Gd-DOTA-L_6_-F2 and Gd-DTPA-L_6_-F2), the steric repulsion between the Gd-complexes weakens the π-π interactions of the phenyl rings. Here we demonstrated that the replacement of the two phenylalanine residues in Gd-DOTA-L_6_-F2 with two 2-naphthylalanine ones allows restoring the stacking interactions. TEM images, at 5.0 mg/mL, show that Gd-2Nal_2_ is able to aggregate in long fibrillary nanostructures, whereas Gd-DOTA-L_6_-F2 remains in an unaggregated form. At the atomic level, the peptide conjugates in the aggregates assume a β sheet secondary structure with an antiparallel orientation of single strands. WAXS/SAXS characterization of the sample, at the solid state, confirms a fibrillary organization with a typical “cross-β pattern”. At the best of our knowledge, this di-naphthylalanine derivative is the most little linear peptide able to give cross-β interactions. Fluorescence studies suggest that Gd-2Nal_2_ peptide derivative begins to self-aggregate at ∼0.1 mg/mL (75 μM), forming stable aggregates when concentration is about 10-fold higher (∼1.0 mg/mL). This CAC value, assessed by fluorescence and proton NMR spectroscopies, is higher than the value (0.076 mg/mL) previously found for tetraphenylalanine Gd-complex Gd-DOTA-L_6_-F4. In the Larmor region, the NMRD profile of Gd-2Nal_2_ (25.0 mg/mL) presents a peak of relaxivity typical of high molecular weight Gd-containing systems. POM images and ^1^HNMR spectra confirmed the incorporation of doxorubicin drug model inside 2Nal based nanostructures. The high values of r1p (12.3 mM^−1^ s^−1^ at 20 MHz) and the capability to encapsulate the doxorubicin anticancer drug suggest a potential use of Gd-2Nal_2_ nanostructures as theranostic systems.

## Experimental section

### Materials and chemicals

All chemical reagents for the peptide synthesis (amino acids, coupling reagents, and the Rink amide 4-methylbenzhydrylamine resin) were available from Calbiochem-Novabiochem (Laufelfingen, Switzerland). Fmoc-Ahoh-OH (Fmoc-21-amino- 4,7,10,13,16,19-hexaoxaheneicosanoic acid) and DOTA (OtBu)_3_-OH were purchased from Neosystem (Strasbourg, France) and Chemateck (Dijon, France), respectively. All other chemical reagents and solvents were purchased by Sigma-Aldrich or Fluka (Bucks, Switzerland) and used as received unless otherwise stated. Purification of peptide conjugates was achieved by employing an HPLC LC8 Shimadzu (Kyoto, Japan) using an UV-detector (lambda-Max Model 481) and a C18-Phenomenex (Torrance, CA) column. Samples were eluted with 0.1% TFA containing H_2_O (A) and 0.1% TFA containing CH_3_CN (B), with a flow rate of 20 mL min^−1^ and a gradient method that moves the B% from 5% to 70% over 30 minutes. Analytical analyses, aimed to identify the products and to assess their purity, were performed using a single quadrupole electrospray ionization, Finnigan Surveyor MSQ (San Jose, CA). Samples were eluted on an analytical C18-Phenomenex column with A and B, with a flow rate of 200 µL min^−1^ and a gradient method that moves the B% from 5% to 70% over 15 minutes.

### Synthesis of peptide derivatives

DOTA-L_6_-F2 and DOTA-L_6_-(2Nal)_2_ were synthesized as previously reported^6, 25, 26^. Briefly, peptide conjugates were synthesized on Rink amide MBHA resin (scale 0.2 mmol, substitution grade 0.65 mmol**/**g). Deprotection of the 9-fluorenylmethoxycarbonyl group (Fmoc) was performed twice for 10 minutes each by treating the peptidyl-resin with a piperidine/N, N-dimethylformamide (70/30, v/v) mixture. The couplings of the amino acids were performed in N, N-dimethylformamide by using 2 equivs. of the amino acid, 2 equivs. of 1-hydroxybenzotriazole (HOBt) and benzotriazol-1-yl-oxy-tris-pyrrolidino-phosphonium (PyBop) and 4 equivs. of diisopropylethylamine (DIPEA) respect to the synthetic scale. After the cleavage from the resin, the crude products were purified by RP-HPLC chromatography and their purity and the identity confirmed by ESI mass spectrometry and ^1^HNMR spectroscopy.

#### DOTA-L_6_-(2Nal)_2_ [2Nal_2_]

^1^H-NMR (CD_3_OD) (chemical shifts in δ, CH_3_OH as internal standard 3.55) = 7.51–7.42 (m, 10 C*H* aromatic), 4.86–4.75 (m, 2H, C*H* Phe α), 3.80 (s, 22H, OC*H*_2_C*H*_2_O), 3.75 (t, 2H, RNHCH_2_C*H*_2_O), 3.70 (s, 6H, R_2_NC*H*_2_COOH), 3.60 (t, 2H, RNHC*H*_2_CH_2_O, 3.45 (s, 16H, R_2_NC*H*_2_C*H*_2_NR_2_), 3.40–3.36 (m, 2H, R_2_NC*H*_2_CONH), 3.16–2.90 (m, 4H, C*H*_2_ Phe β), 2.58 (t, 2H, NHCOC*H*_2_CH_2_O). Retention time, R_t_ = 12.17 min; MS (ESI+): m/z 1132.8 calcd. For C_57_H_80_N_8_O_16_: [M + H^+^] = 1133.8; [M + Na^+^] = 1155.7; [M + 2 H^+^]/2 = 568.1.

### Preparation of gadolinium complexes

Gadolinium complexes were prepared through the addition of GdCl_3_ to the DOTA-functionalized ligands in a molar ratio 1:1 at room temperature and neutral pH. The absence of any residual free Gd^3+^ ions was checked by the well-known UV-Vis method of the orange xylenol^27^; the eventual residual was removed from the solution by adding a further amount of ligand corresponding to the Gd^3+^ excess.

### Preparation of peptide solutions

2Nal_2_ and Gd-2Nal_2_ peptide solutions were prepared by dissolving the lyophilized powder in double distilled water. The concentration of the final solution was determined by absorbance on UV-vis Thermo Fisher Scientific Inc (Wilmington, Delaware USA) Nanodrop 2000c spectrophotometer using a 1.0 cm quartz cuvette (Hellma). The molar absorptivity (ε_280_) for 2Nal_2_ and Gd-2Nal_2_ peptide employed for the spectroscopic measurements was 6070 M^−1^ cm^−1^.

### Fluorescence studies

The determination of CAC (critical aggregate concentration) values was determined as previously described^6^ by the titration of the fluorescent probe ANS (8-anilino-1-naphthalene sulfonic acid ammonium salt)^28, 29^ with the peptide conjugates^30^. Fluorescence spectra of 2Nal_2_ and Gd-2Nal_2_ were recorded at room temperature at several concentrations, exciting samples at 280 nm and recording spectra between 290 and 500 nm.

### NMR experiments

NMR experiments were acquired in the temperature range of 298–303 K on either a Varian Unity Inova 600 MHz spectrometer provided with a cold probe or a 400 MHz Varian instrument provided with a 5-mm triple resonance probe and z-axis pulsed-field gradients. All samples were dissolved in a mixture H_2_O/D_2_O (98% D, Armar Chemicals, Dottingen, Switzerland) 90/10 v/v with a total volume equal to 600 μL. The DOTA-L_6_-F2 was analyzed in the concentration range 0.3 mg/mL (i.e., 0.3 mM)–5.0 mg/mL (i.e., 4.8 mM); for the La-DOTA-L_6_-F2 the examined concentration range was 0.3 mg/mL (i.e., 0.3 mM)–10.0 mg/mL (i.e., 8.5 mM). The 2Nal_2_ compound was analyzed in the concentration range 0.1 mg/mL (i.e., 0.1 mM)–5.0 mg/mL (i.e., 4.4 mM) and La-2Nal_2_ in the range 0.2 mg/mL (i.e., 0.2 mM)–2.5 mg/mL (i.e., 2 mM). 1D [^1^H] experiments along with a series of 2D experiments [(i.e., 2D [^1^H, ^1^H] TOCSY (Total Correlation Spectroscopy)^31^ (70 ms mixing time), 2D [^1^H, ^1^H] NOESY (Nuclear Overhauser Enhancement Spectroscopy)^32^ (300 ms mixing time), and 2D [^1^H, ^1^H] ROESY (Rotating frame Overhauser Enhancement Spectroscopy)^33^ (200 and 250 ms mixing times)] were recorded. 1D spectra were usually acquired with a relaxation delay d1 of 1.5 s and 32–512 scans; 2D experiments were recorded with 16–64 scans, 128–256 FIDs in t1, 1024 or 2048 data points in t2. Assignments of the DOTA-L_6_-F2 and 2Nal_2_-lacking the La (III) ion- were obtained at 1 mM concentration with a canonical protocol^34^ involving comparison of 2D [^1^H, ^1^H] TOCSY^35^ (70 ms mixing time), and 2D [^1^H, ^1^H] ROESY^33^ (200 ms mixing time). Water suppression was achieved by Excitation Sculpting^35^. Spectra were processed with VNMRJ (Varian by Agilent Technologies, Italy) and analyzed with NEASY^36^ comprised in the CARA software package (http://www.nmr.ch/).

### Circular Dichroism

Far-UV CD spectra of the 2Nal_2_ and Gd-2Nal_2_, in 0.5–20 mg/mL concentration range, were recorder on the spectropolarimeter Jasco J-810 equipped with a NesLab RTE111 thermal controller unit. Spectra were recorded between 280 to 195 nm at room temperature, using a standard quartz cell with an path length of 0.1 mm, a scan speed of 10 nm/min a sensitivity of 50 mdeg, a time constant of 16 s and a bandwidth of 1 nm. All the spectra were obtained by averaging three scans, after correction for the solvent and for dilution. Ellipticities were reported as the mean residue ellipticity (MRE).

### Fourier Transform Infrared spectroscopy (FTIR)

Fourier Transform Infrared spectra of 2Nal_2_ and Gd-2Nal_2_ in solution at the concentration of 5.0 mg/mL and at the solid state were collected on the spectrometer Jasco FT/IR 4100 (Easton, MD) in ATR mode, using a Ge single-crystal at a resolution of 4 cm^−1^. All the spectra (100 scans with a rate of 2 mm · s^−1^ against a KBr background) were collected in transmission mode and then converted in emission.

### Congo Red spectroscopic assay

Congo Red (CR) UV-Vis assay was carried out on aforementioned spectrophotometer as previously described^6^. Briefly, 5, 50 and 125 μL of di-naphthylalanine stock solution (20 mg/mL) were added to a CR solution (12, 5 μM) prepared immediately before to use. The solutions containing peptide conjugate incubated with CR were left at room temperature for 30 min and then their UV-Vis spectra were recorded between 400 and 700 nm. The spectrum of CR alone at the same concentration was also recorded in the this spectral region.

### Transmission Electron Microscopy (TEM) images

TEM images were acquired by LaMest (Pozzuoli, Italy) with a FEI TECNAI G12 Spirit-Twin microscope (LaB_6_ source) endowed of a bottom mounted FEI Eagle-4k CCD camera (Eindhoven, The Netherlands), working at 120 kV. The samples for the observation were prepared by placing 10 µL of a solution (5.0 mg/mL) on a holey-carbon coated copper grid (400 mesh). The sample was left to dry at room temperature for 1 hour and stained with a solution (1 wt%) of phosphotungstic acid. Digital images were obtained by FEI Eagle 4 K CCD camera and Xplore 3D software.

### Wide-Angle and Small-Angle X-ray Scattering

Fiber diffraction WAXS and SAXS patterns were recorded from dried fibers prepared according to the well-known method of the stretch frame^13^, in which a droplet (10 μL) of 3 wt% peptide solution, hanged between two wax-coated capillary, was left to dry slowly overnight at room temperature. WAXS and SAXS data were collected at the X-ray MicroImaging Laboratory (XMI-L@b) equipped with a Fr-E+ Super Bright rotating anode copper anode microsource (Cu K_α_, λ = 0.15405 nm, 2475 W), a multilayer focusing optics (Confocal Max-Flux; CMF 15-105) and a three-pinhole camera (Rigaku SMAX-3000)^37^. For WAXS data collection an image plate (IP) detector with 100 µm pixel size was placed at 8.5 cm from the sample and calibrated by means of the Si NIST standard reference material (SRM 640b). For SAXS data collection a Triton 20 gas-filled photon counter detector with ~200 µm pixel size was placed at 2.2 m from the sample and calibrated by means of silver behenate. A detailed description of the XMI-L@b performances can be found in Altamura *et al.* ^37^ and Sibillano *et al*.^38^.

### Water proton relaxation measurements

Measure of R_1_ (longitudinal water proton relaxation rates) of Gd-2Nal_2_ were carried out on a Stelar Spinmaster spectrometer (Pavia, Italy) operating at 0.5 T which corresponds to 21.5 MHz Proton Larmor Frequency. R_1_ (1/T_1_) values were measured at 298 K by mean of the standard inversion-recovery technique. For the measure of the proton 1/T_1_ NMRD (Nuclear Magnetic Resonance Dispersion) profiles, a fast field-cycling Stelar relaxometer, operating over a continuum of magnetic field strengths from 0.00024 to 0.47 T (corresponding to 0.01–20 MHz proton Larmor frequencies) was used. The temperature was set at 298 K. The relaxometer is computer controlled and the absolute uncertainty is 1/T_1_ ± 1%. Additional data points were obtained on a Stelar Spinmaster spectrometer coupled to an electromagnet tunable at different Larmor Frequencies between 21.5 and 70 MHz. Experimental temperature control was obtained with an air-flow heater (Stelar VTC-91) endowed of a copper constantan thermocouple (uncertainty 0.1 °C). A previously reported relaxometric method^39^ was used for the determination of the concentrations of the solutions used for the relaxometric characterization. The conventional Solomon-Bloembergen-Morgan theory was used for fitting of experimental data.

### Doxorubicin loading and leakage

A DOX solution (1 · 10^−4^ M) was placed in the cuvette and titrated with small amount of Gd-2Nal_2_ peptide conjugate at 50 mg/mL. The fluorescence of each sample was monitored on Jasco Model FP-750 spectroflurophotometer as above described. Excitation wavelength was settled at 480 and UV-Vis spectra were collected between 490 and 700 nm. At the end of the titration the DOX filled Gd-2Nal_2_ aggregates were 10-fold diluted and the DOX fluorescence was monitored up to 72 h. The extent of the DOX leakage was calculated as follows: *Leakage* (*%*) = 100 (F_i_ − F_0_)/(F_100_ − F_0_), where F_i_ represents the level of fluorescence measured for each time point, whereas F_100_ and F_0_ are the fluorescence levels before and after the peptide addition in cuvette, respectively.

### Doxorubicin loading of by ^1^HNMR and polarized optical microscopy (POM)

To explore encapsulation of doxorubicin in 2Nal_2_, a sample at a concentration of 2.5 mg/mL was used and 1D [^1^H] NMR spectra were recorded in absence and presence of doxorubicin (concentration going from 0.05 to 1 mg/mL). POM images of Gd-2Nal_2_ at 20 mg/mL and of Gd-2Nal_2_/DOX (20 and 1 mg/mL, respectively) in water solution were obtained observing samples with AZ100 microscope (Nikon) between crossed polars.

## Electronic supplementary material

Supplementary info
