# Supplementary material for: Cross-beta nanostructures based on dinaphthylalanine Gd-conjugates loaded with doxorubicin
Source: Sci Rep. 2017 Mar 22;7:307. doi: 10.1038/s41598-017-00332-3 (PMC5428233; doi:10.1038/s41598-017-00332-3)
Supplement: Supplementary file 1 — Supplementary info [file 41598_2017_332_MOESM1_ESM.doc]

**Supporting Information**

**Cross-beta nanostructures based on dinaphthylalanine Gd-conjugates loaded with doxorubicin**

Carlo Diaferia,^a^ Eliana Gianolio,^b^ Teresa Sibillano,^c^ Flavia Anna Mercurio,^d^ Marilisa Leone,^d^ Cinzia Giannini,^c^ Nicole Balasco,^d^ Luigi Vitagliano,^d^ Giancarlo Morelli,^a^ and Antonella Accardo*^a^

^a^Department of Pharmacy, Research Centre on Bioactive Peptides (CIRPeB), University of Naples “Federico II”, Via Mezzocannone 16, 80134-Naples, Italy

^b^Department of Molecular Biotechnologies and Health Science, University of Turin, Via Nizza 52, 10125-Turin, Italy

^c^Institute of Crystallography (IC), CNR, Via Amendola 122, 70126-Bari, Italy

^d^Institute of Biostructure and Bioimaging (IBB), CNR, via Mezzocannone 16, 80134-Naples, Italy

| **A)** |
| --- |
| 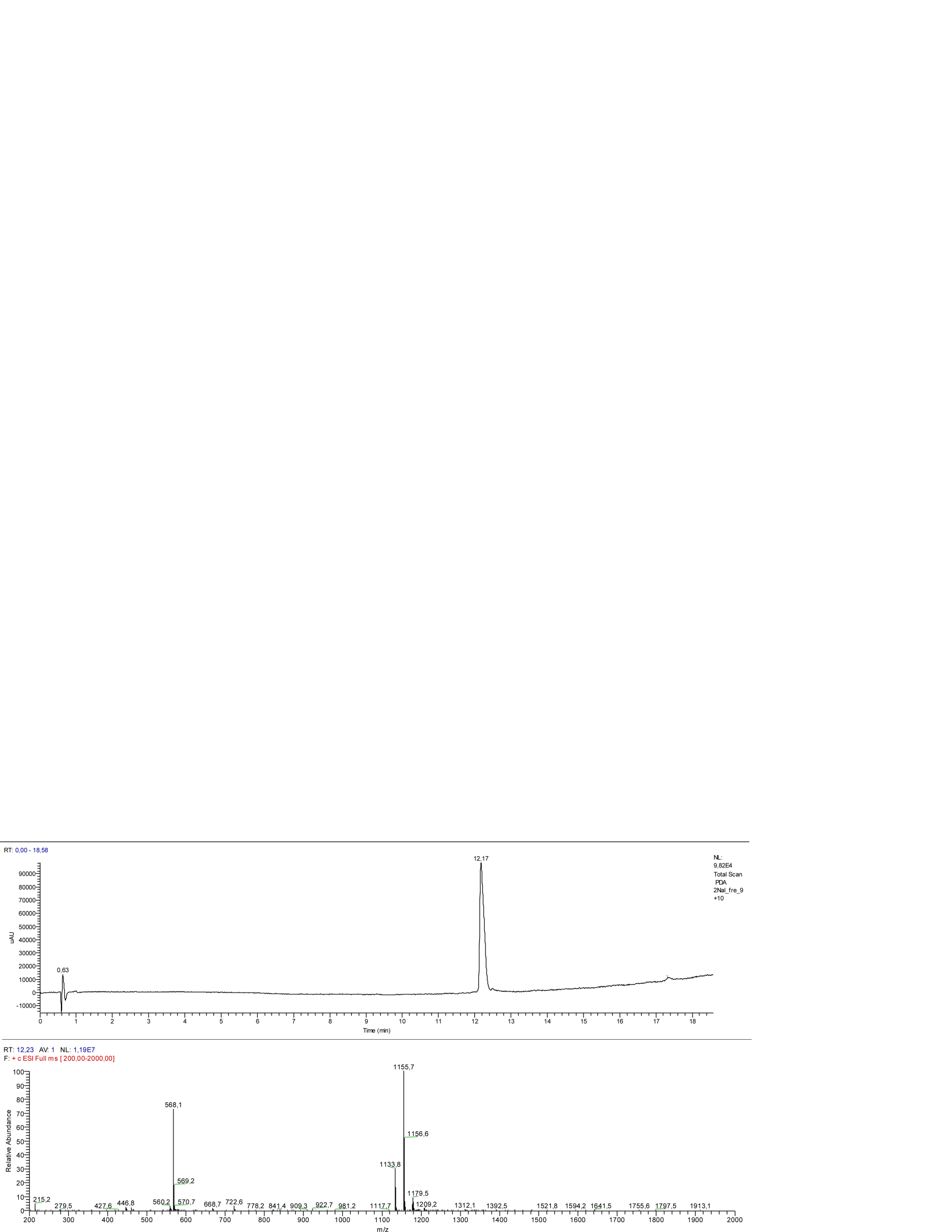 |
| B) |
| 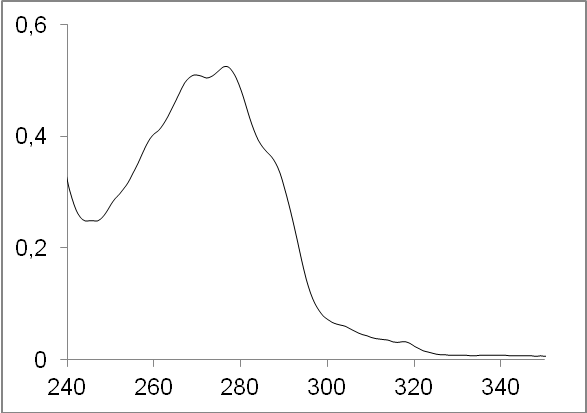 |

**Figure S1:** Physicochemical characterization of 2Nal_2_ : A) LC-MS profile; B) UV-Vis spectrum

T= 0


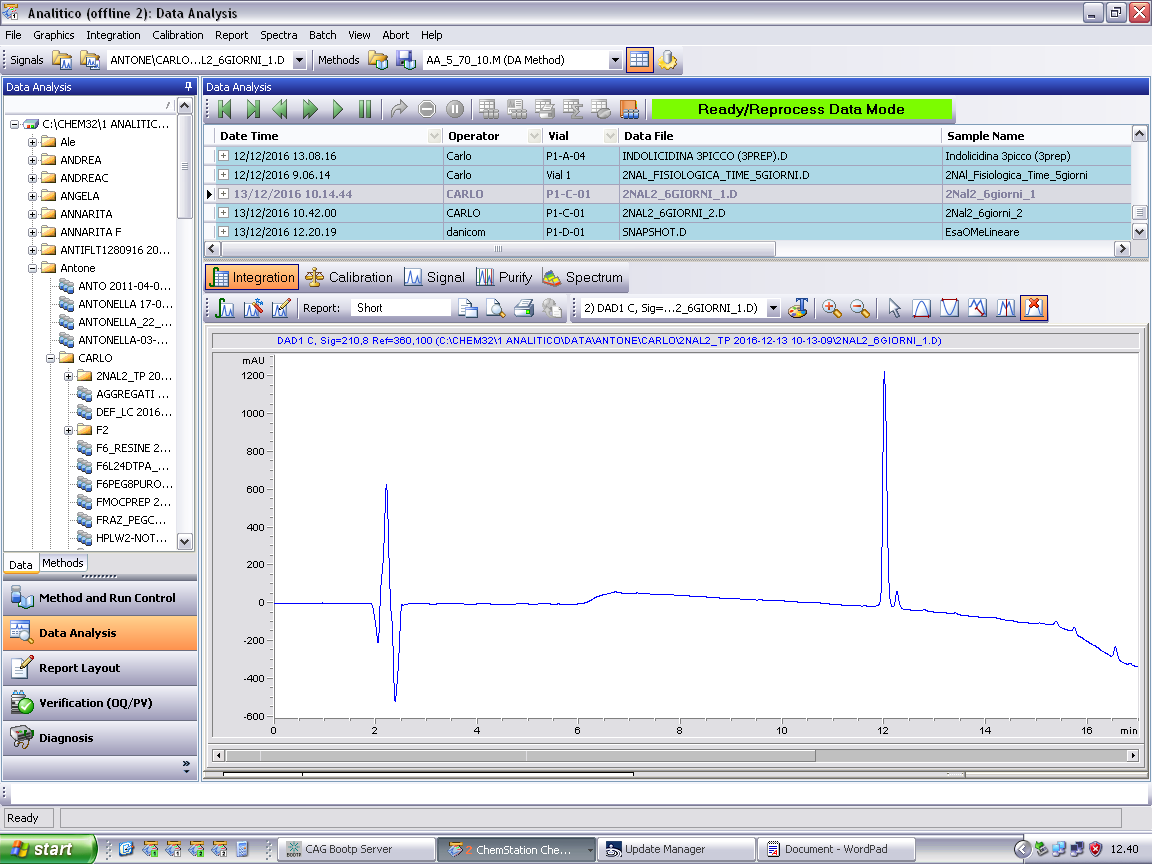


T= 240 h


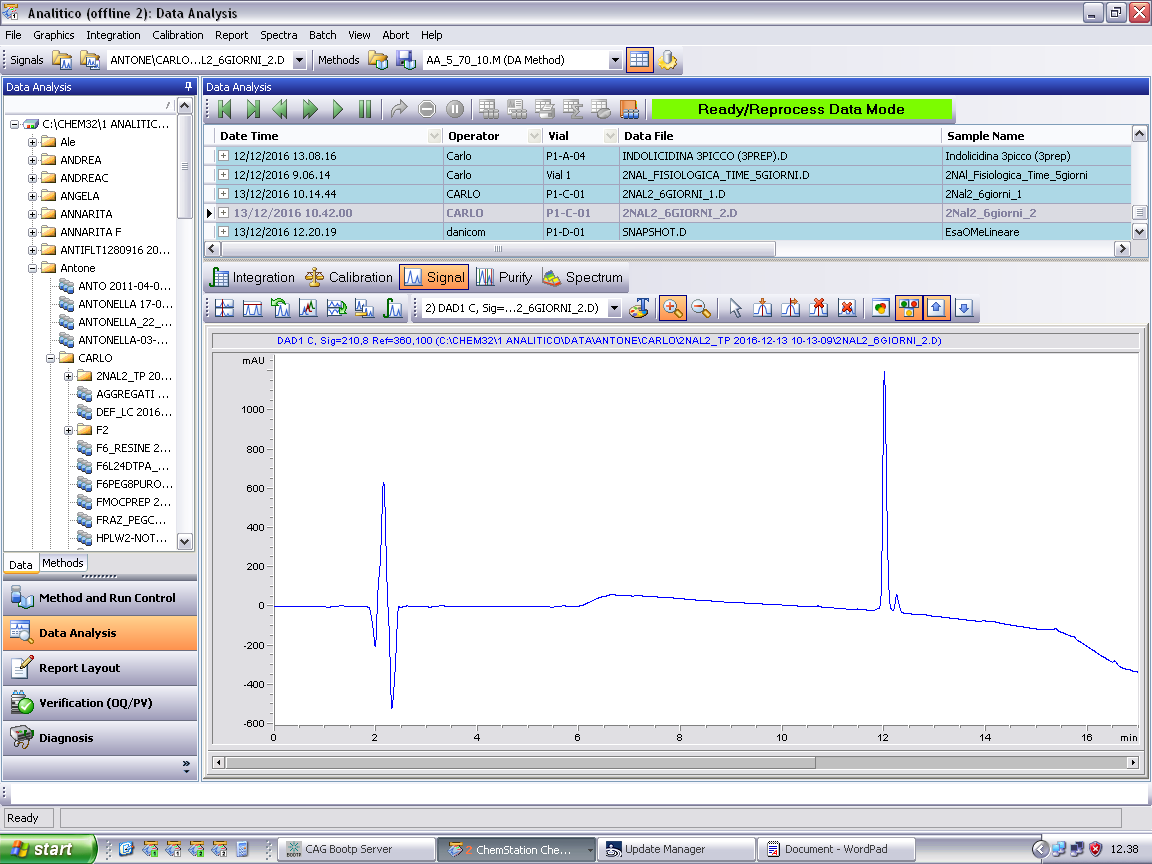


**Figure S2:** Reverse phase HPLC profiles of Gd-2Nal_2_ in 10 mM phosphate buffer 0.9 wt.% NaCl at pH 7.4 at time T=0 and T=240 h.

***
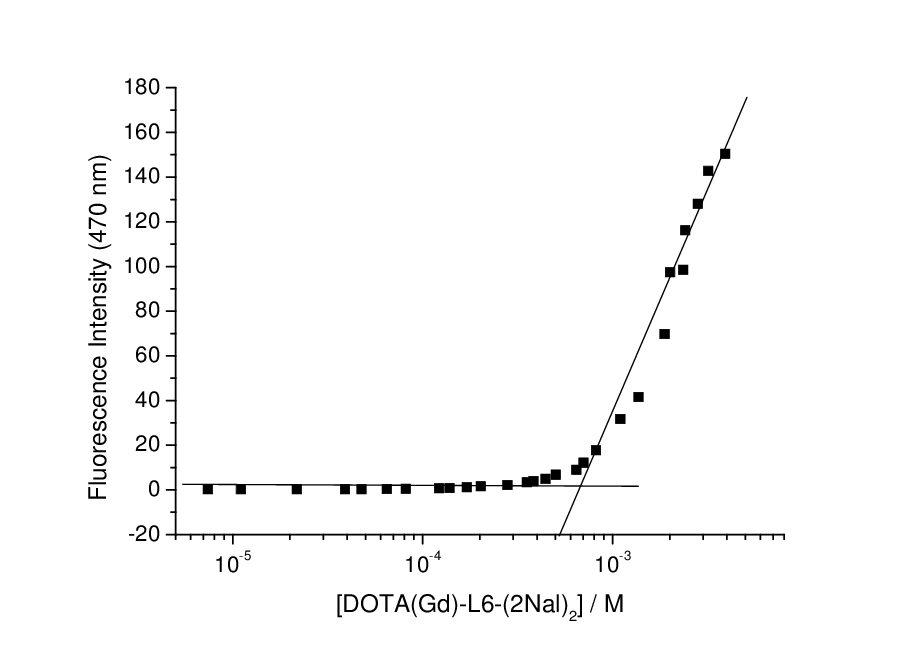
***

**Figure S3:** Fluorescence intensity emission of ANS fluorophore at 470 nm *vs.* concentration of 2Nal_2_ gadolinium complex. CAC value is established from the break point.

|  |  |
| --- | --- |

**Figure S4:** **Left panel** A) 1D ^1^H NMR spectra recorded for DOTA- L6-F2 at 5 mg/mL (4.8 mM), 2.5 mg/mL (2.4 mM), 1.2 mg/mL (1.2 mM), 0.6 mg/mL (0.6 mM), 0.3 mg/mL (0.29 mM). B) 1D ^1^H NMR spectra recorded with La-DOTA- L6-F2 at 10 mg/mL (8.5 mM), 5 mg/mL (4.3 mM), 2.5 mg/mL (2.1 mM), 1.2 mg/mL (1 mM), 0.6 mg/mL (0.5 mM), 0.3 mg/mL (0.26 mM). **Right panel** A) 1D ^1^H NMR spectra recorded for 2Nal_2_ at 5 mg/mL (4.4 mM), 2.5 mg/mL (2.2 mM), 1.2 mg/mL (1.1 mM), 0.6 mg/mL (0.5 mM), 0.3 mg/mL (0.3 mM), 0.1 mg/mL (0.1 mM). B) 1D ^1^H NMR spectra recorded with La-2Nal_2_ at 2.5 mg/mL (2 mM), 1.2 mg/mL (0.9 mM), 0.8 mg/mL (0.6 mM), 0.4 mg/mL (0.3 mM), 0.2 mg/mL (0.2 mM).

**Table S1.** Chemical shifts of F2-L6-DOTA (1 mM concentration) at 298 K.

| **F2-L6-DOTA** | | | | |
| --- | --- | --- | --- | --- |
|  | HN | Hα | Hβ | Others |
| F1 | 8.12 | 4.51 | 2.94-2.87 | Aromatics  7.14  7.31 |
| F2 | 8.17 | 4.52 | 3.12-2.88 | Aromatics  7.24   7.34  CONH2 7.06-7.00 |
| L6 | NHCO**CH2** 2.43  NHCOCH2**CH2** 3.62  NH**CH2CH2** 3.58, 3.34  **NH**CH2CH2 8.05  O**CH2CH2**O 3.52, 3.57 | | | |
| DOTA | 3.77, 3.33, 3.11 | | | |

**Table S2.** Chemical shifts of 2Nal_2_ (1mM concentration) at 298 K.

| **2Nal_2_** | | | | |
| --- | --- | --- | --- | --- |
|  | HN | Hα | Hβ | Others |
| NAL1 | 7.97 | 4.53 | 2.94 | Aromatics: 7.45, 7.18, 7.75 |
| NAL2 | 8.15 | 4.63 | 3.32-3.00 | Aromatics: 7.67, 7.38,  7.85  CONH2 7.10-6.89 |
| L6 | NHCO**CH2** 2.19, 2.12  NHCOCH2**CH2** 3.40, 3.30  NH**CH2CH2** 3.52, 3.30  **NH**CH2CH2 7.98  O**CH2CH2**O 3.52, 3.57 | | | |
| DOTA | 3.69, 3.24, 3.03 | | | |

**Figure S5.** A) TOCSY 70 (left panel) and ROESY 200 (right panel) spectra of DOTA-L_6_-F2 (1 mM concentration). B) TOCSY 70 (left panel) and ROESY 250 (right panel) NMR spectra of 2Nal_2_ (1 mM concentration).

**
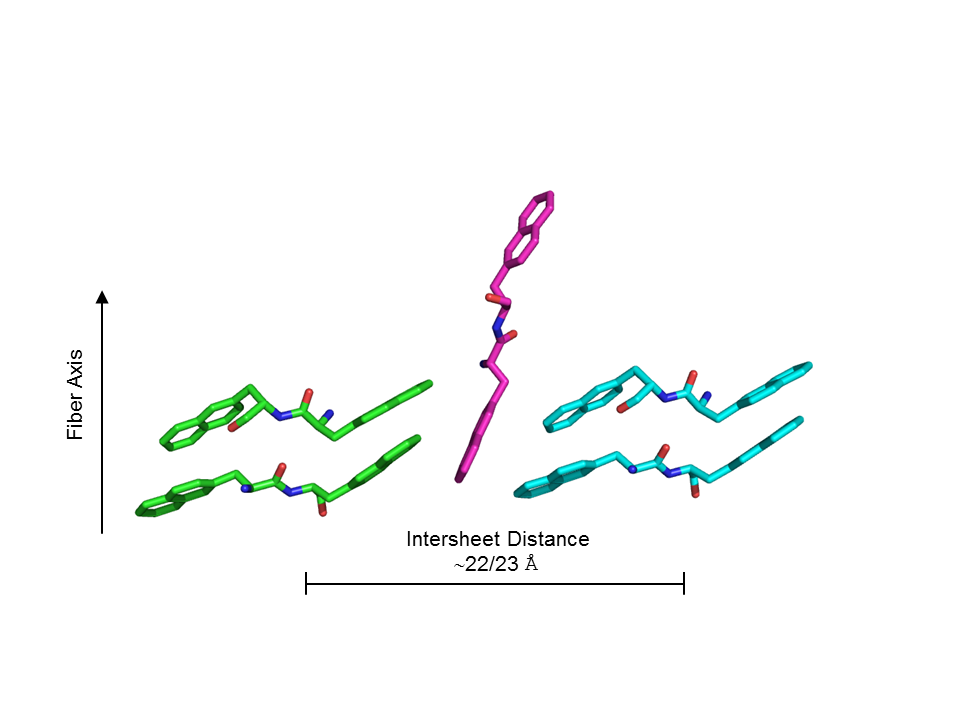
**

**Figure S6.** A three-dimensional model of the possible insertion way of a Nal side chain within the dry interface of two facing β-sheets.

| **A)** | 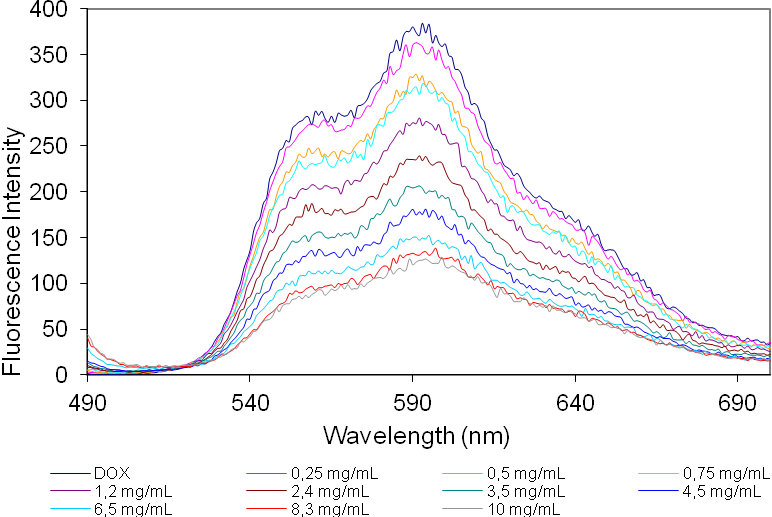 |
| --- | --- |
| **B)** | 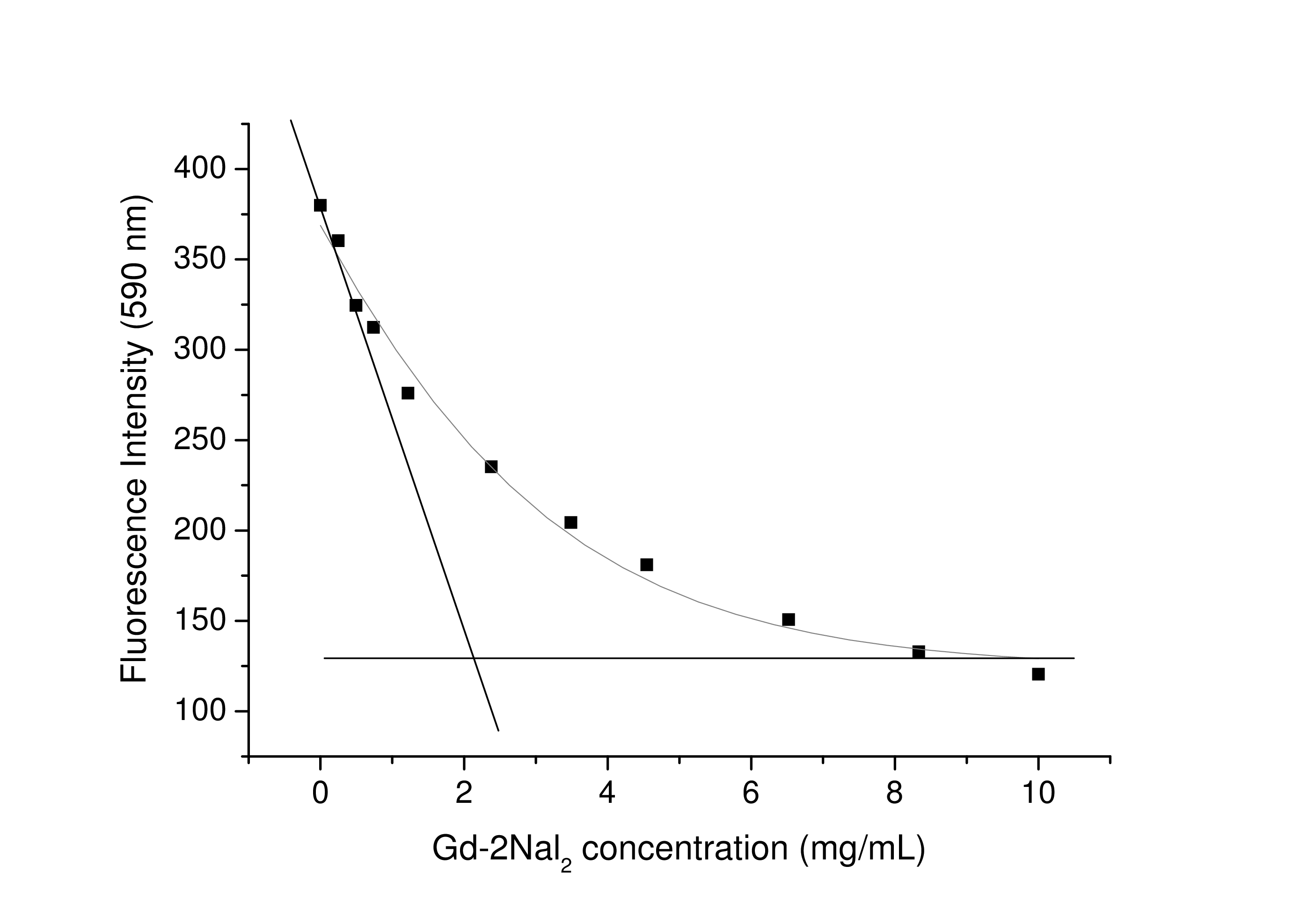 |

**Figure S7: A)** Fluorescence spectra of 1·10^-4^ M DOX (58 μg/mL) solution alone and after the addition of growing amounts of Gd-2Nal_2_ peptide conjugate (from 0.25 to 10.0 mg/mL). The progressive decrease of the DOX fluorescence intensity is due to the stacking of the anthracycline with the naphthylalanine ring. The excitation wavelength of DOX was settled at 480 nm and spectra recorded between 490 and 700 nm. B) Fluorescence intensity in the maximum at 590 nm as function of the Gd-2Nal_2_ concentration reported in mg/mL.


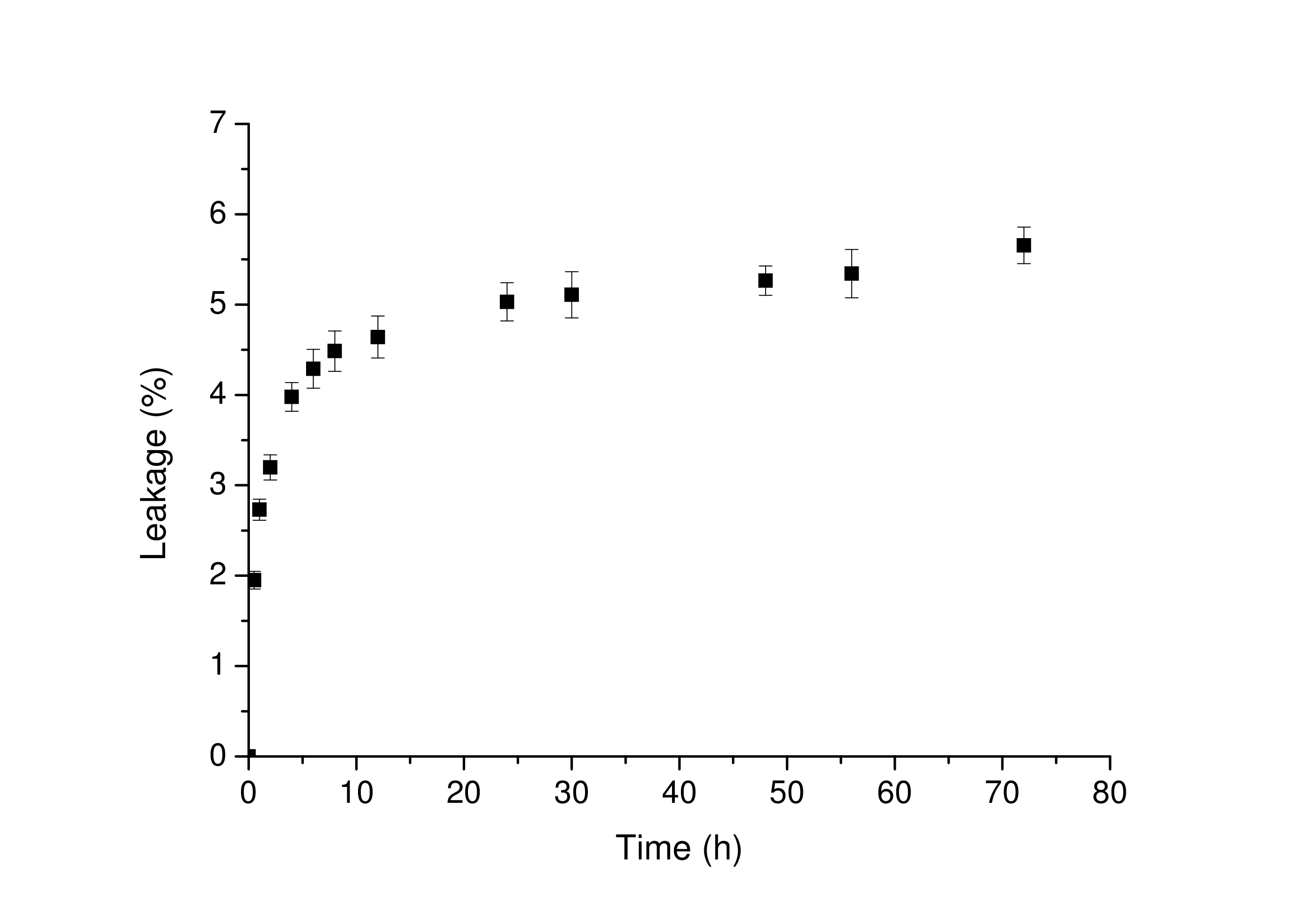


**Figure S8:** Leakage (%) of DOX from Gd-2Nal_2_ aggregates over the time.
